# Mutual Self-Regulation of d-Electrons of Single Atoms and Adjacent Nanoparticles for Bifunctional Oxygen Electrocatalysis and Rechargeable Zinc-Air Batteries

**DOI:** 10.1007/s40820-023-01022-8

**Published:** 2023-02-11

**Authors:** Sundaram Chandrasekaran, Rong Hu, Lei Yao, Lijun Sui, Yongping Liu, Amor Abdelkader, Yongliang Li, Xiangzhong Ren, Libo Deng

**Affiliations:** 1 College of Chemistry and Environmental Engineering, Shenzhen University, Shenzhen, 518060 People’s Republic of China; 2 College of Chemistry and Bioengineering, Guilin University of Technology, Guilin, 541004 People’s Republic of China; 3 Shenzhen Key Laboratory of Special Functional Materials, Shenzhen Engineering Laboratory for Advanced Technology of Ceramics, College of Materials Science and Engineering, Guangdong Research Center for Interfacial Engineering of Functional Materials, Shenzhen University, Shenzhen, 518060 People’s Republic of China; 4 Shanghai Key Laboratory for R&D and Application of Metallic Functional Materials, Institute of New Energy for Vehicles, School of Materials Science and Engineering, Tongji University, Shanghai, 201804 People’s Republic of China; 5 Department of Design and Engineering, Faculty of Science & Technology, Bournemouth University, Poole, BH12 5BB Dorset UK

**Keywords:** Cyclodextrin, CD-MOF, Single-atom catalyst, ORR/OER, Zinc-air battery

## Abstract

Rechargeable zinc-air batteries (ZABs) are a promising energy conversion device, which rely critically on electrocatalysts to accelerate their rate-determining reactions such as oxygen reduction (ORR) and oxygen evolution reactions (OER). Herein, we fabricate a range of bifunctional M–N–C (metal-nitrogen-carbon) catalysts containing M–N_*x*_ coordination sites and M/M_*x*_C nanoparticles (M = Co, Fe, and Cu) using a new class of γ-cyclodextrin (CD) based metal–organic framework as the precursor. With the two types of active sites interacting with each other in the catalysts, the obtained Fe@C-FeNC and Co@C-CoNC display superior alkaline ORR activity in terms of low half-wave (*E*_1/2_) potential (~ 0.917 and 0.906 V, respectively), which are higher than Cu@C-CuNC (~ 0.829 V) and the commercial Pt/C (~ 0.861 V). As a bifunctional electrocatalyst, the Co@C-CoNC exhibits the best performance, showing a bifunctional ORR/OER overpotential (Δ*E*) of ~ 0.732 V, which is much lower than that of Fe@C-FeNC (~ 0.831 V) and Cu@C-CuNC (~ 1.411 V), as well as most of the robust bifunctional electrocatalysts reported to date. Synchrotron X-ray absorption spectroscopy and density functional theory simulations reveal that the strong electronic correlation between metallic Co nanoparticles and the atomic Co-N_4_ sites in the Co@C-CoNC catalyst can increase the d-electron density near the Fermi level and thus effectively optimize the adsorption/desorption of intermediates in ORR/OER, resulting in an enhanced bifunctional electrocatalytic performance. The Co@C-CoNC-based rechargeable ZAB exhibited a maximum power density of 162.80 mW cm^−2^ at 270.30 mA cm^−2^, higher than the combination of commercial Pt/C + RuO_2_ (~ 158.90 mW cm^−2^ at 265.80 mA cm^−2^) catalysts. During the galvanostatic discharge at 10 mA cm^−2^, the ZAB delivered an almost stable discharge voltage of 1.2 V for ~ 140 h, signifying the virtue of excellent bifunctional ORR/OER electrocatalytic activity.

**Supplementary Information:**

The online version contains supplementary material available at 10.1007/s40820-023-01022-8.

## Highlights

- A new class of γ-cyclodextrin based metal–organic framework -derived strategy was used to fabricate highly active M–N-C catalysts containing both single-atom sites and nanoparticles.
- The obtained Co@C-CoNC exhibits remarkable bifunctional oxygen reduction (ORR)/oxygen evolution reactions (OER) performance, which further delivered a high power density and excellent cyclic stability in rechargeable zinc-air batteries.
- Density functional theory calculation suggests that the mutual self-regulation of d-electron density of both Co NP and Co SAC conjointly reduce the reaction energy barriers and thus boost the ORR/OER kinetics through their fast adsorption/desorption ability of reaction intermediates.

**Supplementary Information:**

The online version contains supplementary material available at 10.1007/s40820-023-01022-8.

## Graphical abstract

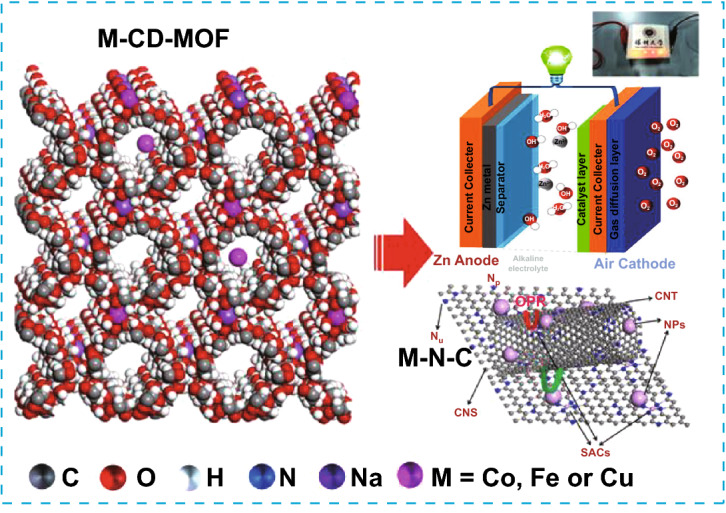

## Introduction

Rechargeable zinc-air batteries (ZABs), which convert chemicals into electrochemical energy through oxygen reduction and evolution reactions (ORR and OER), are an important technique with great promise in the future energy system due to their high energy density and excellent safety. The ORR/OER reactions are generally kinetically sluggish and require a large overpotential, resulting in unsatisfying power density and poor durability of this device [1–7]. These reactions are conventionally accelerated by precious metal-based catalysts such as Pt (for ORR), Ru, and Ir (for OER). However, the poor stability, low tolerance to poison, and high cost have been hindering the large-scale applications of precious metal-based ZABs [8, 9]. In addition, Pt and Ru/Ir-based catalysts alone do not efficiently catalyze ORR and OER simultaneously [2, 10, 11]. The combination of these electrocatalysts, such as Pt/C + RuO_2_ or Pt/C + Ir/C, serves as the first-generation bifunctional electrocatalysts in rechargeable ZABs [1, 12]. However, due to the dissolution and redeposition of catalysts during the catalytic process, this combination technique usually leads to mutual interference, which restricts their extensive use. Hence, developing low-cost, highly efficient, and durable non-precious metal-based bifunctional electrocatalysts is of utmost importance and is a central theme for realizing cost-effective high-performance rechargeable ZABs.

Recent studies revealed the important role of atomic-scale transition metals in various electrocatalysts, such as Fe/OES [13], Fe–N/GNs [14], Co–N–C [15], Zn-N_4_-C [16] and Cu-SA/SNC [17] for ORR; and Co-P SAC/MWCNT [18], Fe_2_/Co_1_-GNCL [19], and S, N-Fe/N/C-CNT [20] for OER. In this context, carbonization of metal–organic frameworks (MOFs) is one of the most prominent methods to prepare single-atom catalysts (SACs), which works on the basis that the metal coordination center in the crystalline MOF is immobilized and thus aggregation of metal species is inhibited during carbonization due to the rigid crystal structure. Owing to the highly exposed active sites, superior electrical conductivity, and excellent durability, a variety of SACs have been prepared using this method [9, 12, 21–24]. For example, Fe-SAC derived from ZIF-8 showed an impressive performance for ORR, and a Co-SAC derived from Zn/Co bimetallic MOF outperformed the commercial Pt/C for ORR [25, 26]; a dual-atom catalyst (Fe_2_/Co_1_-GNCL) derived from a unique MOF with abundant ordered aromatic ring arrays outperformed the commercial IrO_2_ for OER [19]. However, the performance of the bifunctional oxygen electrocatalysts relying solely on SACs, characterized by the parameter Δ*E*, is still too large such that a high overpotential is needed (Δ*E* is the difference between the OER potential to reach a current density of 10 mA cm^−2^ (*E*_10_) and the ORR half-wave potential of the limiting current density (*E*_1/2_)). For example, the Δ*E* of typical SACs, such as Fe–N_*x*_–C (Δ*E* ~ 0.920 V) [27], CoSAs-NGST (Δ*E* ~ 0.90 V) [10], and dual single atoms of Ni-N_4_/GHSs/Fe-N_4_ (Δ*E* ~ 0.790 V) [28] are still unsatisfying. To this end, Li et al. demonstrated that further incorporating metal-containing nanoparticles (NPs) could remarkably enhance the bifunctionality due to the synergistic effects of single atoms and small NPs [29]. The small NPs not only improve the graphitization degree of carbon supports but are also beneficial to the multi-step electron transfer process and mitigate the structural failure of active parts during the discharge–charge cycle [30]. This was then validated by Lu et al. through the fabrication of Co-SAs/SNPs@NC from pyrolysis of Zn/Co-ZIFs [31]. However, a systematical comparison of the bifunctionality of the most prominent transition metal-based catalysts such as Fe, Co, and Cu-based catalysts, is still lacking.

Cyclodextrin (CD) is an oligosaccharides derived from starch and consists of 6–8 glucose units interconnected by 1,4-R-glucosidic bonds. They are crystalline, homogeneous, and non-hygroscopic substances with truncated cone-shaped molecules [32]. It has a macrocycle cavity with the outer hydrophilic surface of cones consisting of hydroxyl groups, while the inner hydrophobic cavities are covered by glycosidic oxygen and C–H units, forming a unique hydrophilic outer surface and a hydrophobic inner cavity [32, 33]. The numerous hydroxyl groups could coordinate with alkaline metals to form a new family of “edible MOF” [34]. The coordination confinement and free space in CD-based MOF provide an excellent platform for constructing metallic active sites for electrocatalysts. However, this new type of MOF has not been used as the precursor for preparing electrocatalysts so far.

In this work, we prepared CD-based MOF using γ-CD as the ligand and sodium (Na) as the coordination center. A series of transition metals were then impregnated into the MOF. A portion of the metal ions replace the original Na sites in the crystallite whereas the other ions are freely adsorbed in the framework. These M-loaded CD-MOFs (M = Co, Fe, and Cu) were then carbonized, giving rise to single-atomic sites located adjacent to metal-containing NPs (*i.e.,* M/M_*x*_C) in the carbonaceous matrix (denoted as M@C-MNC). Due to the interaction between NPs and M–N_*x*_ sites, these electrocatalysts demonstrated excellent bifunctional electrocatalytic activity. Particularly, rechargeable ZAB based on the Co-CD-MOF derived catalyst (Co@C-CoNC) delivered a power density up to 162.8 mW cm^−2^. Density functional theory (DFT) calculation revealed that the mutual self-regulation of the d-electron density of both metallic NPs and the adjacent M-N_4_ SAC sites in M@C-MNC conjointly reduces the reaction energy barriers and thus boosts the bifunctional ORR/OER kinetics through their rapid adsorption/desorption ability of reaction intermediates. Most importantly, the order of catalytic activity among the three types of transition metals was quantitatively revealed, *i.e.,* the downshift d-band center of Fe-SACs in the Fe@C-FeNC with respect to SACs in bare Fe–N_*x*_ is the highest and thus a highest ORR activity was observed, whereas the negative shift of the d-band center of Co-NPs in the Co@C-CoNC with respect to bare NPs in Co@C is the highest which led to the best OER activity.

## Experimental

### Reagents

All reagents were purchased from Sigma-Aldrich and Macklin Reagent Co., China. All chemicals, including γ-Cyclodextrin (γ-CD), sodium hydroxide (NaOH), Cobalt(II) nitrate (Co(NO_3_)_2_·6H_2_O), Iron nitrate (Fe(NO_3_)_3_·9H_2_O), Copper(II) nitrate (Cu(NO_3_)_2_·H_2_O), methanol (MeOH), polyethylene glycol (PEG 2000 or PEG 20,000), dicyandiamide (C_2_H_4_N_4_) and ethanol, were used without further purification.

### Synthesis of CD-MOF and M-CD-MOFs

To synthesize the MOFs, a mother solution (45 mL) was first prepared by mixing γ-CD (750 mg) and NaOH (360 mg) in pure water (15 mL) with the pre-addition of 25 mL methanol. Then 750 mg of PEG was added quickly followed by continuous stirring for 30 min, followed by adding 5 mL methanol. The resultant solution was sealed into a Teflon-lined stainless steel autoclave and hydrothermally treated at 110 °C for 12 h. The solid product was obtained by repeated centrifuging with water/ethanol solution and dried at 60 °C for 12 h. The obtained product was denoted as CD-MOF. To prepare the M-CD-MOFs, the metal precursors of 0.02 M of cobalt nitrate, iron nitrate, and copper nitrate solution were added into the mother solution (*i.e.,* before the addition of PEG) with the same procedure as CD-MOF to attain Co-CD-MOF, Fe-CD-MOF, and Cu-CD-MOF, respectively.

### Fabrication of MDC and M–N-C Catalysts

To prepare the M–N-C materials, a crucible containing dicyandiamide was placed in a tube furnace in the upstream of another crucible containing M-CD-MOF (the mass ratio between the two was 1:1) and treated at 800 °C for 2 h with a ramp rate of 5 °C min^−1^ in flowing Ar gas in a tube furnace, yielding black powders that were stirred in 2 M HCl solution for 12 h to remove the undesired metal particles. After thorough washing and drying at 60 °C for 12 h, the second pyrolysis was conducted at 800 °C for 2 h with a ramp rate of 5 °C min^−1^ under Ar gas in a tube furnace yielding the M–N–C catalysts, which were denoted as Co@C-CoNC, Fe@C-FeNC, and Cu@C-CuNC catalysts. Similarly, the as-prepared CD-MOF was annealed at 500 °C for 2 h with a ramp rate of 5 °C min^−1^ in flowing Ar gas in a tube furnace to get the MDC (i.e., bare CD-MOF derived carbon).

## Results and Discussion

### Characterization of the Morphology, Structure, and Composition

**Fig. 1:**
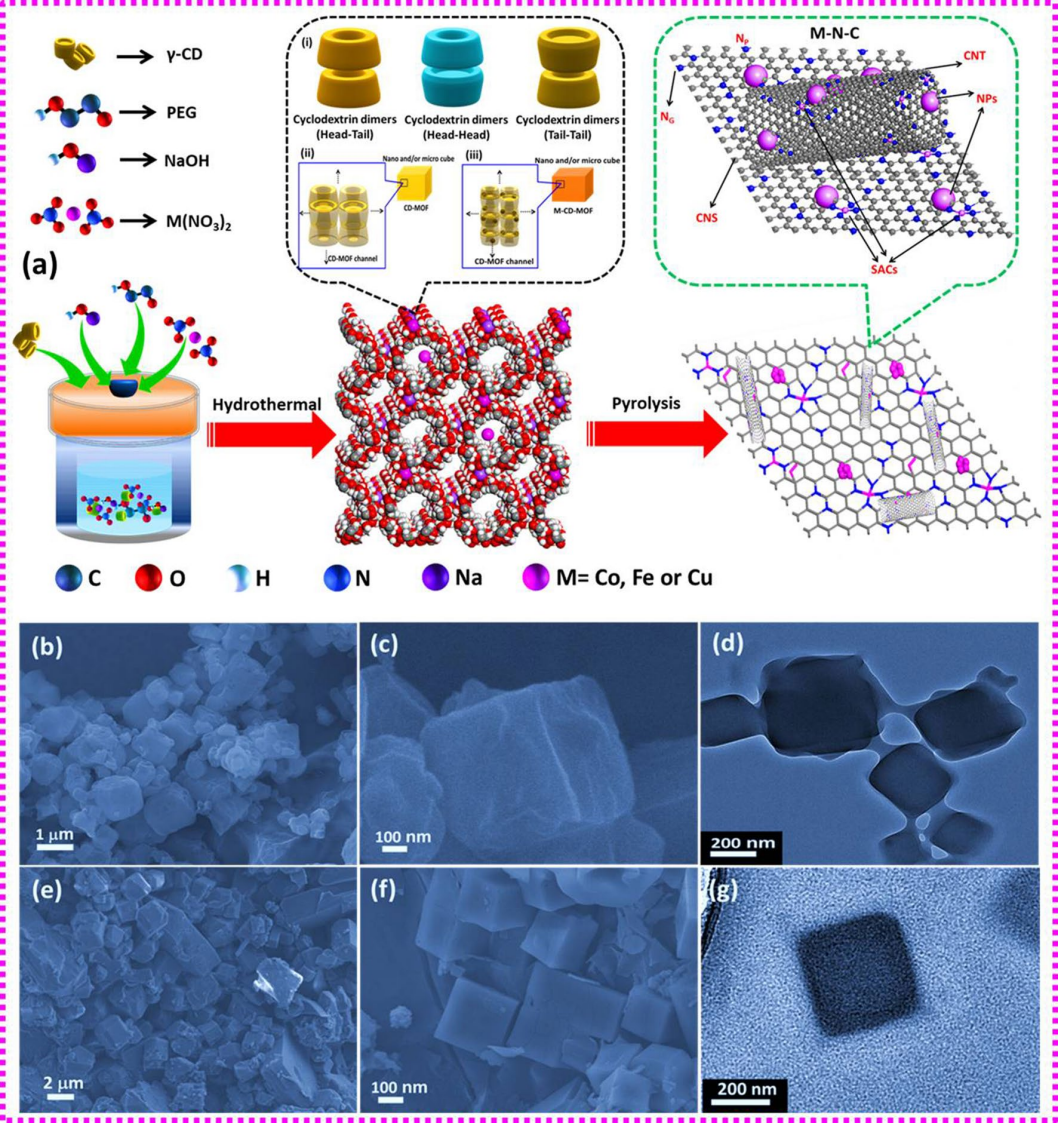
**a** Schematic of the preparation of the bare CD-MOF, M-loaded CD-MOFs, and the M–N–C catalysts, in which the insets are: (i) three possible orientations for CD dimers (HH, HT, and TT); (ii-iii) Proposed formation mechanism based on molecular dynamics notion and experimental results for CD-MOF and M-CD-MOF nanostructures through the γ-CD_channel_ growth; FE-SEM and TEM images of: **b-d** bare CD-MOF and **e–g** Co-CD-MOF

The strategy for the synthesis of bare CD-MOF, metal-impregnated MOF (M-CD-MOF), and the carbonized M–N–C catalysts are shown in Fig. 1a. Single crystals of CD-MOF and M-CD-MOF were also prepared via a vapour diffusion method (Scheme S1). As shown in Fig. S1, the single crystal of bare CD-MOF exhibited a triclinic structure of C_96_H_164_Na_2_O_82_ with lattice parameters of *a* = 15.1513(5), *b* = 16.8018(5), and *c* = 17.0695(6) Å. The microscopic images of bare CD-MOF (Fig. 1b–d), Co-CD-MOF (Fig. 1e–g), Fe-CD-MOF (Fig. S2), and Cu-CD-MOF (Fig. S3) suggest that the CD-MOF and Co-CD-MOF samples are consisted of uniform cubic of nano- and micro-structure with even surface and an average size of ~ 200 nm to 1 μm, while the Cu-CD-MOFs possess cubic morphology with nano/micropores on the surface. Recently, both experimental and molecular dynamics/molecular mechanics theory suggested that there are three possible orientations between the CD units, including the head-to-head (H–H), tail-to-tail (T–T), and head-to-tail (H–T) structures (Fig. 1a) [35]. The complex formed between γ-CD and their guests including alkali and/or metal ions was revealed by single-crystal X-ray diffraction (XRD) analysis (Fig. S1). The cubic morphology for CD-MOF, Co-CD-MOF, and Cu-CD-MOF may conceivably be formed through the controlled packing of CD molecules in the crystal lattice based on the γ-CD channel (*i.e.* channel type structure of γ-CD_channel_) formation method, where γ-CD molecules were stacked together in an H–H or H–T alignment to form a column in the crystal [36]. When the metal precursor of Co or Cu was replaced by Fe, the as-prepared Fe-CD-MOF shows a hexagonal morphology, where the long length was grown through the two-stage γ-CD_channel_ formation strategy. During the first stage, structural alignment of the H–H step forms the short-length hexagonal channels, and then the long-length structure was formed by further extension of γ-CD_channel_ through either HT or TT [36]. These structures suggest that the alkali and transition metal cations including Na, Co, Fe, and Cu may be located among the nearest γ-CD molecules in the γ-CD_channel_ or between the γ-CD_channel_ in the γ-CD structures. Field emission scanning electron microscopy (FE-SEM) and transmission electron microscopy (TEM) inspection signify that shallow truncated cone arrangements of host CDs are highly preserved to anchor various guest molecules. In addition, as shown in Fig. 1a, the metal ions may replace the coordination center atom of Na in the host lattice of CD-MOF [32]. Upon carbonization, the metal ions at the coordination center can form catalytically active single-atom sites due to the confinement of the ligand, whereas those adsorbed in the pores of the CD-MOF could result in NPs via self-aggregation. These two types of metal species in the carbonaceous matrix are crucial for realizing high-performance bifunctional oxygen electrocatalysis.

It has been shown previously that the incorporation of guest molecules can lead to an increase in the distance between the γ-CD channels consistent with (200) planes (*i.e.,* powder X‐ray diffraction (XRD) peak at ~ 7.5°) in the microstructures, which can result in the bulky cubic and long-length structures [36, 37]. The XRD patterns of our nano-/micro-cubes and long-length CD-MOFs showed a fairly resilient peak at ~ 7.3° characteristic of the γ-CD_channel_ (Fig. S4) [36, 37]. It suggests that all the prepared MOFs could be composed of channel-type arrangements of γ-CD. Thermogravimetric analysis (TGA) characterization indicates the carbon yield is approximately 20% at 800 °C for the bare CD-MOFs and M-CD-MOFs (M = Co, Fe, or Cu) (Fig. S5). In addition, nitrogen-doped carbons were also prepared by the carbonization of the M-CD-MOFs in the presence of dicyandiamide (as a nitrogen source) at 800 °C as shown in Fig. 1, yielding Co@C-CoNC, Fe@C-FeNC and Cu@C-CuNC catalysts, respectively (for more details, *see* Experimental Section).

**Fig. 2:**
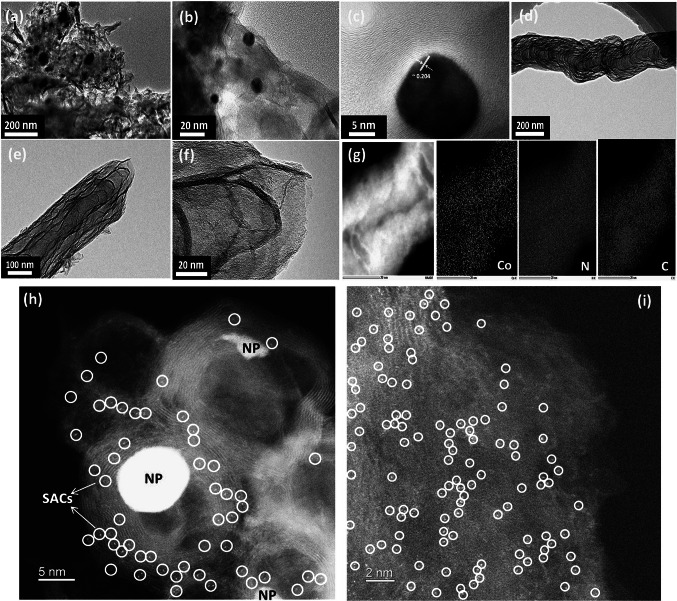
TEM images of Co@C-CoNC catalyst at different locations and different magnifications: **a-f** shows the co-existence of small-sized nanoparticles (NPs) and N–C/CNT hybrids of Co@C-CoNC catalyst; **g** EDS mapping; HAADF-STEM images of: **h** shows Co SACs adjacent to Co NPs and **i** represents the isolated Co SACs in a carbonaceous hybrid matrix of Co@C-CoNC catalyst

Figure 2 shows the FE-SEM, TEM, high-angle annular dark-field scanning TEM (HAADF-STEM) images, and energy dispersive spectroscopy (EDS) maps of the M–N–C catalysts. From the FE-SEM images of M–N–C catalysts (Figs. S6-S8), it can be seen that the matrices are consisted of both porous graphitic carbon and carbon nanotubes (CNTs with diameters of ~ 80–150 nm) in Co- and Fe-CD-MOF-derived carbons, whereas CNTs are absent in the Cu-CD-MOF-derived carbon. CNTs are formed only in Fe- and Co-derived carbons due to a relatively high solubility of carbon in these metals, which allows the dissolved carbon to precipitate out from the *in situ* formed NPs to form CNTs [38]. During the high-temperature treatment of M-CD-MOF, the metal ions in the M-CD-MOF are reduced to a certain extent, in which the free migration gives rise to metal/metal carbide NPs while the strong confinement leads to isolated atomic metal. The TEM and HAADF-STEM images for Co@C-CoNC (Fig. 2), Fe@C-FeNC (Fig. S7), and Cu@C-CuNC (Fig. S8) show that uniform and highly isolated single atoms of Co, Fe and Cu with their corresponding M/M_*x*_C NPs are distributed in the carbonaceous matrices. Furthermore, the HR-TEM image of Fe@C-FeNC reveals that these CNTs are composed of crystalline graphitic planes, and the lattice fringes with an inter-planar distance of ∼0.335 nm correspond to the (002) plane of the graphitic carbon (Fig. S7j).

To gain more insights into the dispersion state of metal species, we conducted HAADF‐STEM inspection into the M–N–C catalysts. A large number of uniformly dispersed spots (marked by yellow circles), which are assigned to single Co, Fe, and Cu atoms, respectively, can be seen in Fig. 2h, i for Co@C-CoNC, Fig. S7k-l for Fe@C-FeNC, and Fig. S8i-j for Cu@C-CuNC. On the other hand, several NPs with diameters of ~ 10–20 nm also appear in the HAADF-STEM images of these M–N-C catalysts. EDS mapping results as shown in Figs. 2g and S7-S8 confirm that these NPs are the aggregation of metals, and they are surrounded by metal ions and N atoms on the atomic scale. These results suggest the co-existence of M–N_*x*_ and M/M_*x*_C NPs in M–N–C catalysts. Such a kind of atomic environment would allow for the interaction of NPs and M–N_*x*_ moieties. Recent reports by Zhao et al. [8] and Li et al. [29] suggest that the interaction between NPs and M–N_*x*_ moieties could significantly enhance the catalytic performance. The contents of metals such as Co, Fe, and Cu in the Co@C-CoNC, Fe@C-FeNC, and Cu@C-CuNC catalysts were determined to be ~ 2.59, ~ 2.95 and ~ 1.23 wt%, respectively, using inductively coupled plasma optical emission spectrometry (ICP-OES).

**Fig. 3:**
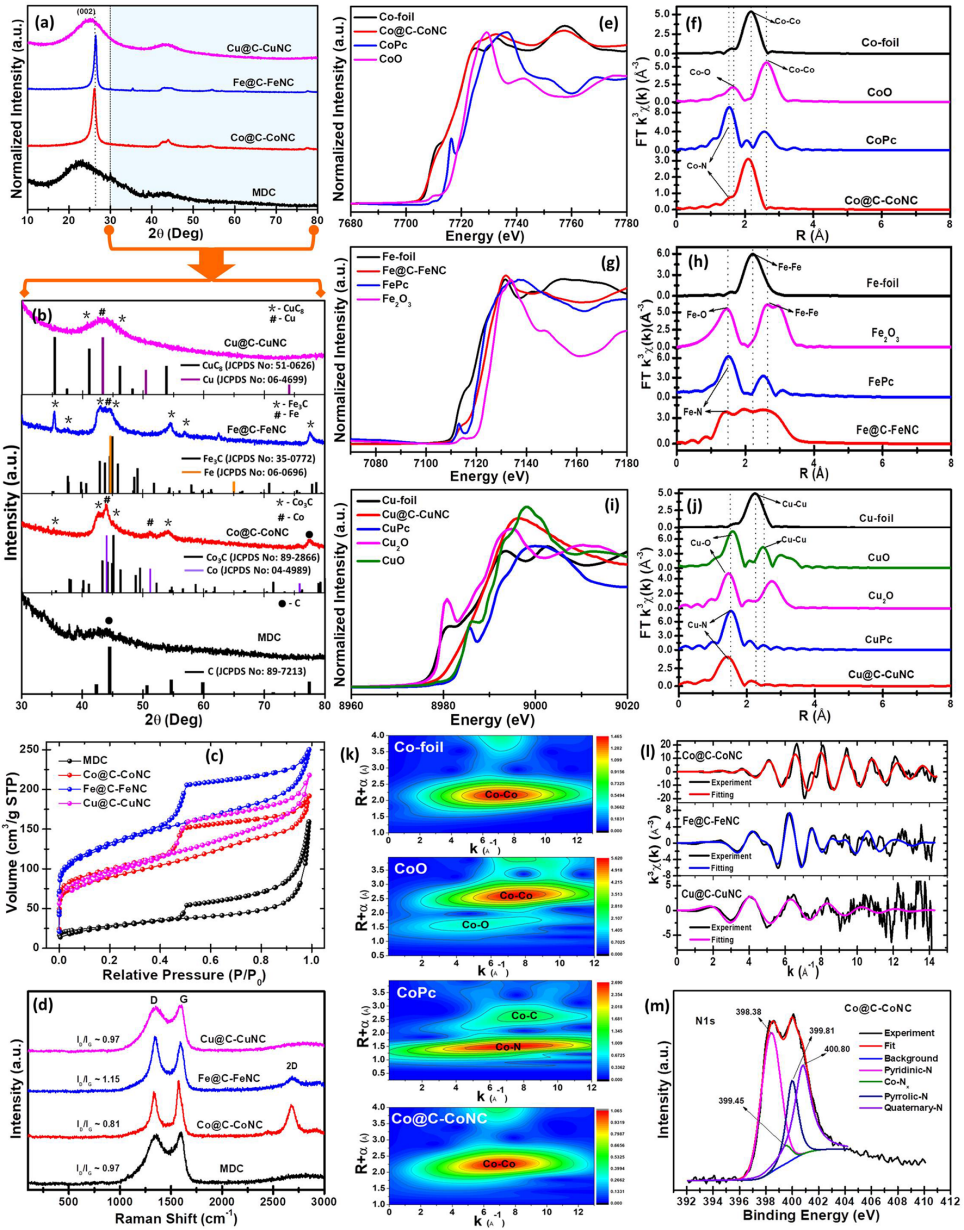
**a** XRD patterns and their enlarged view in the range from 30–80° are shown in **b**. **c** N_2_ adsorption–desorption isotherms and **d** Raman spectra of MDC, Co@C-CoNC, Fe@C-FeNC, and Cu@C-CuNC catalysts; Normalized K-edge XANES and K-edge Fourier transform-EXAFS in R space: **e–f** for Co@C-CoNC, **g-h** for Fe@C-FeNC, and **i-j** for Cu@C-CuNC catalysts with their corresponding metal foils, metal oxides, and metal phthalocyanines (MPc) as references; **k** EXAFS wavelet transforms of Co@C-CoNC catalyst with its corresponding reference samples; **l** EXAFS *k* space fitting curves of the M–N–C catalysts, and **m** N 1*s* core-level XPS spectrum of Co@C-CoNC catalyst

The XRD patterns of M–N–C catalysts are shown in Fig. 3a. The peak at 26.63° is related to the (0 0 2) plane of graphitic crystallites. This peak is particularly intense and narrow in Co@C-CoNC and Fe@C-FeNC catalysts, indicating a higher crystallinity and structural order in these materials than in Cu@C-CuNC and MDC, which is in line with the TEM inspection. The enlarged view of the XRD patterns at high angles (*i.e.,* 2θ value from 30°–80°, Fig. 3b) suggests that the sharp and broad peaks are indexed to metallic Co (JCPDS No: 04–4989) and Co_3_C (JCPDS No: 89–2866) for Co@C-CoNC, metallic Fe (JCPDS No: 06–0696) and Fe_3_C (JCPDS No: 35–0772) for Fe@C-FeNC, and metallic Cu (JCPDS No: 06–4699) and CuC_8_ (JCPDS No: 51–0626) for Cu@C-CuNC. Moreover, the N_2_ adsorption–desorption isotherm curves for M–N-C catalysts displayed I/IV‐type isotherms representing the features of micro/mesoporous materials (Fig. 3c). All the M–N–C samples such as Co@C-CoNC, Fe@C-FeNC and Cu@C-CuNC exhibited a highly porous feature, with surface areas of ~ 320.99, 464.23, and 340.68 m^2^ g^−1^, respectively. The structural evolution was also revealed by Raman spectroscopy (Fig. 3d). In addition to the characteristic D- and G- bands in all the pyrolytic carbons, a well-defined 2D band was also seen in Co@C-CoNC and Fe@C-FeNC, suggesting a higher degree of graphitization in these two carbons due to the catalytic graphitization effect of Co and Fe, which is consistent with the TEM observation that CNTs are formed in these samples. A high degree of graphitization is known to be beneficial for electrocatalysis [39].

To scrutinize the chemical environments of M–N–C catalysts, X-ray absorption fine structure (XAFS) measurements were performed. The absorption edge of X-ray absorption near-edge structure (XANES) spectroscopy and K-edge Fourier transform- extended X-ray absorption fine structure (EXAFS) in R-space for Co@C-CoNC, Fe@C-FeNC and Cu@C-CuNC catalysts and their references are shown in Figs. 3e–j and S9-S10. The K-edge XANES spectra of M–N-C catalysts designate the absorption energy values at near-edges were found within the range of reference metal foils (Fig. 3e, g, and i). Figure 3f, h, and j reveals peaks at ~ 1.42 Å for Co@C-CoNC, 1.39 Å for Fe@C-FeNC, and 1.40 Å for Cu@C-CuNC attributing to the distance of M–N moieties due to an N shell surrounding metal ions for all the M–N–C catalysts, confirming the existence of the M–N_*x*_ coordination in these materials [15, 40]. There are additional peaks at ~ 2.2 and 2.9 Å for the M–N–C catalysts attributing to M–M and M–C distances, respectively, indicating the co-existence of metal (M) and their carbide (M_*x*_C) NPs [41–43]. Furthermore, EXAFS wavelet transforms (EXAFS-WT) were performed to identify the atomic dispersion of the metal atoms in the M–N–C catalysts with their corresponding reference samples (Figs. 3k and S11-S12). Specifically, for the Co@C-CoNC catalyst, only one maximum intensity for Co–Co in the k space of EXAFS-WT was observed in Co@C-CoNC, denoting the metal-rich nature of Co@C-CoNC. In addition, least-square EXAFS curve-fitting suggests that the N-coordination number of Co, Fe, and Cu in Co@C-CoNC, Fe@C-FeNC and Cu@C-CuNC is ~ 4, 3 and 5, respectively (Table S1). Figures 3, S13-S15 and Table S2 show the X‐ray photoelectron spectra (XPS) and the chemical compositions of the M–N-C catalysts. Figures S13-S15 show the core-level spectra of Co 2*p*, Fe 2*p*, and Cu 2*p*, designating that Co@C-CoNC, Fe@C-FeNC, and Cu@C-CuNC contain both metal Co (Co^0^) and mixed valences of Co^2+^/Co^3+^, metal Fe (Fe^0^) and mixed valences of Fe^2+^/Fe^3+^, and metal Cu (Cu^0^) and mixed valences of Cu^+^/Cu^2+^ states, respectively. As shown in Fig. 3m, the core-level N 1*s* spectra show the contribution of different N-species including pyridinic-N, pyrrolic-N, quaternary-N, and the metal-N (M–N_*x*_) coordination at ~ 398.38, 399.81, 400.80, and 399.45 eV, respectively, for Co@C-CoNC [44, 45]. Figures S14-S15 demonstrate the existence of different N-species in Fe@C-FeNC and Cu@C-CuNC catalysts, respectively. By taking all characterization results into account, we can conclude that all the M–N–C catalysts are comprised of NPs and M–N_*x*_ sites.

### Electrocatalytic Performance

**Fig. 4:**
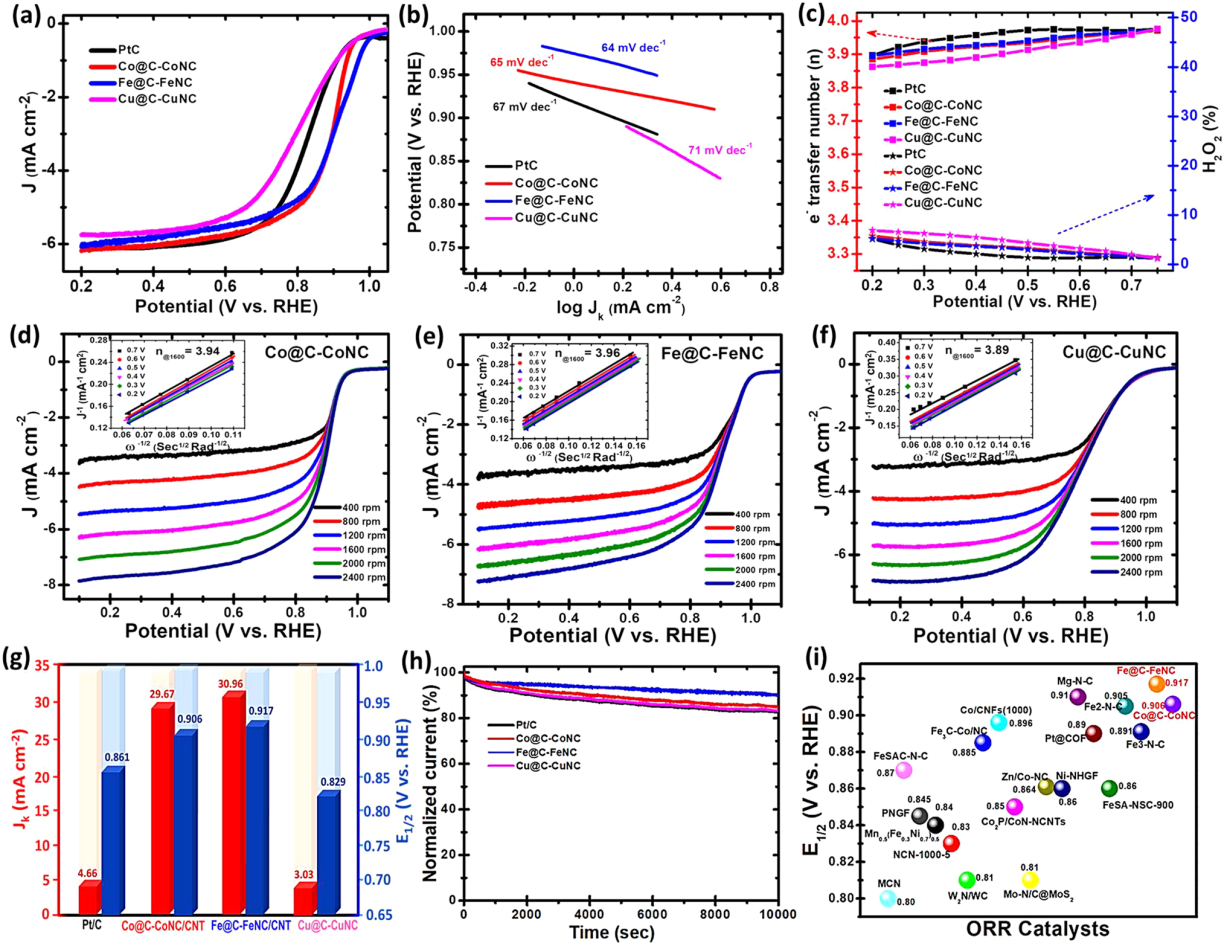
**a** ORR polarization curves and **b** their corresponding Tafel plots for Co@C-CoNC, Fe@C-FeNC, Cu@C-CuNC, and the benchmark Pt/C catalysts at a rotation rate of 1600 rpm in O_2_‐saturated 0.1 M KOH electrolyte; **c** Electron-transfer number (*n*) (top) and H_2_O_2_ yield (bottom) *vs.* Potential; The ORR polarization curves at different rotating rates of: **d** for Co@C-CoNC, **e** for Fe@C-FeNC and **f** for Cu@C-CuNC, in which the insets are K − L plots and electron-transfer numbers; **g** Comparison of *J*_k_ at 0.85 V and *E*_1/2_ of Co@C-CoNC, Fe@C-FeNC and Cu@C-CuNC catalysts with Pt/C; **h** Durability tests at 0.67 V (at 1600 rpm); and **i** Comparison of the ORR activity of the present study with recently reported catalysts

To assess the electrocatalytic performance of the as-prepared catalysts towards ORR, cyclic and linear sweep voltammograms (LSVs) were recorded on a rotating disk electrode (RDE) at 1600 rpm in 0.1 M KOH electrolyte at a scan rate of 10 mV s^−1^ (Figs. S16-S19 and Fig. 4). As shown in Fig. 4a, Fe@C-FeNC and Co@C-CoNC exhibit superior ORR activity with a more positive half-wave (*E*_1/2_) potential of 0.917 and 0.906 V (all the potential values are expressed relative to the reversible hydrogen electrode, RHE), which are higher than that of the benchmark Pt/C (0.861 V) and Cu@C-CuNC (0.829 V). The wide current plateau from 0.7 to 0.2 V represents a diffusion-controlled process analogous to the efficient four-electron (4e^−^) dominated ORR pathway. The ORR onset potential at 1600 rpm was ~ 1.025 V for Fe@C-FeNC, and this value is more positive than that of Co@C-CoNC (0.988 V), Cu@C-CuNC (0.976 V) and Pt/C (0.974 V). In addition, the diffusion current (*J*_*d*_) at 0.2 V is about –6.18, –6.07, –5.75, and –6.07 mA cm^–2^ for Co@C-CoNC, Fe@C-FeNC, Cu@C-CuNC and Pt/C, respectively. Among all these M–N–C catalysts, Fe@C-FeNC shows the best ORR activity, while the Co@C-CoNC is slightly inferior. Furthermore, the superior catalytic ORR kinetics of Fe@C-FeNC and Co@C-CoNC is evident by a smaller Tafel slope of 64 and 65 mV dec^−1^ in the low overpotential region (Fig. 4b), which is lower than that of Pt/C (67 mV dec^−1^).

Rotating ring disk electrode (RRDE) tests signify that in the potential range 0.2–0.75 V, the H_2_O_2_ yields are in the range of 1.24–5.25%, 1.42–5.74%, 1.18–6.88% and 1.21–5.13%, corresponding to an electron-transfer number of 3.90–3.98, 3.88–3.97, 3.86–3.98 and 3.90–3.98 for Fe@C-FeNC, Co@C-CoNC, Cu@C-CuNC and Pt/C, respectively *(*Fig. 4c). It shows that all the M–N–C catalysts undergo an efficient catalytic process via a ~ 4e^−^ ORR pathway. RDE measurements at different rotation rates were further carried out to evaluate the ORR pathway (Fig. 4d–f). The Koutechy–Levich (*K–L*) plots of Fe@C-FeNC, Co@C-CoNC and Cu@C-CuNC manifest good linearity over the potential from ~ 0.7 to 0.2 V, offering first-order ORR reaction kinetics (inset in Fig. 4d–f). As shown in Fig. 4g, the kinetic current densities (*J*_k_) are ~ 30.96 and 29.67 mA cm^−2^ at 0.85 V for Fe@C-FeNC and Co@C-CoNC, which are about ~ 6.64 and 6.36 folds higher than that of the commercial Pt/C (4.66 mA cm^−2^), respectively. In addition, the Fe@C-FeNC displayed excellent long-term activity (only ∼18% activity loss after 10,000 s of operation) and excellent LSV reproducibility after long-term durability (Figs. 4h and S20-S22). The electrochemical active surface area (ECSA) was also assessed by the double-layer capacitance (Cdl), and the Cdl values are 13.4 and 11.9 mF cm^−2^ for Co@C-CoNC and Fe@C-FeNC (Fig. S23). In addition, electrochemical impedance spectroscopy (EIS) was used to evaluate the electronic conductivity of M–N–C catalysts. As shown in Fig. S23, Co@C-CoNC and Fe@C-FeNC displayed a smaller-charge transfer resistance representing their rapid charge transfer efficiency. Furthermore, the Co@C-CoNC and Fe@C-FeNC catalysts revealed a high methanol cross-over tolerance and were sensitive to cyanide (SCN^–^) poison in O_2_-saturated 0.1 M KOH (Figs. S24-S25). However, under the acidic condition, the M–N–C catalysts revealed a slightly lower ORR activity compared to Pt/C (Fig. S26). Remarkably, the electrocatalytic ORR activity and kinetics of Fe@C-FeNC and Co@C-CoNC in alkaline conditions outperform most of the recently reported non-precious catalysts (Fig. 4i and Table S3), such as Co-SAs/SNPs@NC [31], FeCo SAs@Co/N-GC [46], Fe SA-NSC-900 [45], CoNP@FeNC [47], Co/CoFe@NC [48], and Fe_3_C-Co/NC [49].

**Fig. 5:**
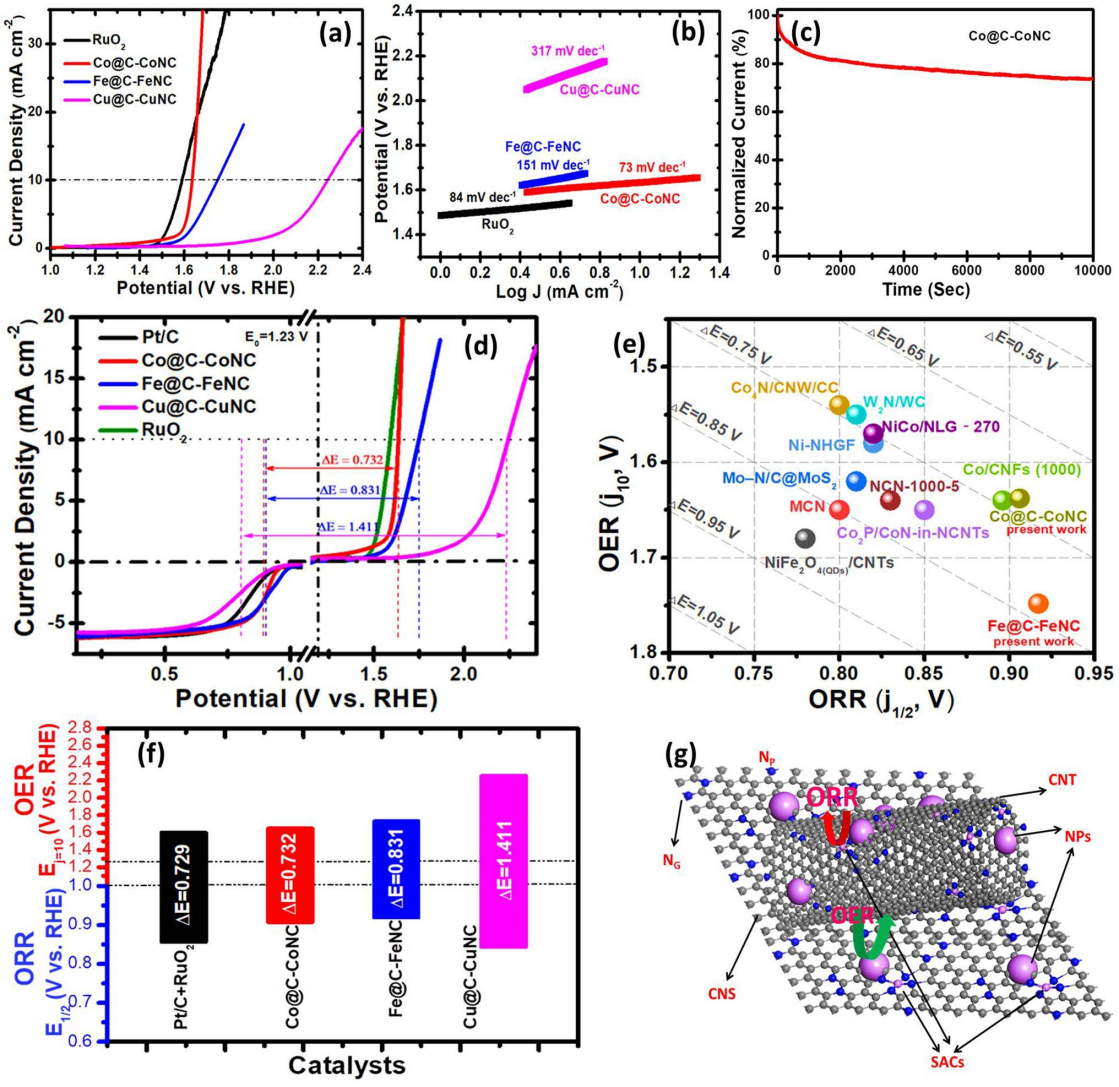
**a** OER polarization curves and **b** their corresponding Tafel plots of Co@C-CoNC, Fe@C-FeNC, and Cu@C-CuNC, and RuO_2_ catalysts in O_2_‐saturated 0.1 M KOH solution; **c** Durability tests of Co@C-CoNC catalyst in O_2_‐saturated 0.1 M KOH solution at a constant overpotential of 0.43 V (at 1600 rpm); **d** Electrocatalytic bifunctional LSV polarization curves of Co@C-CoNC, Fe@C-FeNC, and Cu@C-CuNC catalysts tested in O_2_‐saturated 0.1 M KOH electrolytes; **e** Comparison of the bifunctional activity with the literature data; **f** Summarized features and realizing the best robust alkaline OER, ORR, and bifunctional ORR/OER catalysts of the present study; **g** Proposed active sites for ORR and OER in the M–N–C catalysts

The OER activity of the as-prepared bare MDC, M-MDC samples and M–N–C catalysts is also evaluated in O_2_-saturated 0.1 M KOH electrolytes (Figs. S27 and Fig. 5). As shown in Fig. 5a, the OER polarization curves for Co@C-CoNC suggested much better OER activities than Fe@C-FeNC and Cu@C-CuNC, and are similar to RuO_2_. To attain a current density of 10 mA cm^−2^, Co@C-CoNC, Fe@C-FeNC, Cu@C-CuNC and RuO_2_ required overpotentials of ~ 0.408, 0.518, 1.010 and 0.360 V, with Tafel slopes of ~ 73, 151, 371 and 84 mV dec^−1^, respectively (Fig. 5b). The OER activity of Co@C-CoNC and Fe@C-FeNC is comparable or even higher than most of the robust OER catalysts reported to date (Table S4), such as Meso-CoNC@GF [50], Co_2_P/CoN-in-NCNTs [51], and Fe–N_*x*_–C [27]. In addition, the Co@C-CoNC catalyst also showed good stability, with ~ 21% activity loss after 10,000 s of operation (Figs. 5c and S28). The XPS spectra of M–N-C after the durability test signify that the catalysts display almost identical chemical compositions with negligible degradation (Figs. S29-S31 and Table S5).

As shown in Fig. 5d, the application of the M–N–C catalysts for both ORR and OER was explored. The overall bifunctional activity is measured by the difference between OER and ORR metrics, *i.e.,* Δ*E* = *E*_*j*=10_ − *E*_1/2_, where a smaller Δ*E* indicates a higher activity. Remarkably, the Co@C-CoNC exhibits a Δ*E* of 0.732 V, which is similar to the combination of benchmark catalysts Pt/C + RuO_2_ (0.729 V) and much lower than that of Fe@C-FeNC (0.831 V) and Cu@C-CuNC (1.411 V), as well as most of the robust bifunctional catalysts reported to date (Fig. 5e, f and Table S6), such as CoS/CoO@NGNs [52], Co-N_4_/NC [53], Fe–N_*x*_–C [27], FeN_*x*_-PNC [54], Meso-CoNC@GF [50], Co_2_P/CoN-in-NCNTs [51], and Ni–N_4_/GHSs/Fe–N_4_ [28]. This enhancement is associated with the interaction between M/M_*x*_C NPs and M–N_*x*_ active sites (Fig. 5g) which are further discussed in detail in the Density Functional Theory (DFT) section.

### Performance of Rechargeable ZABs

**Fig. 6:**
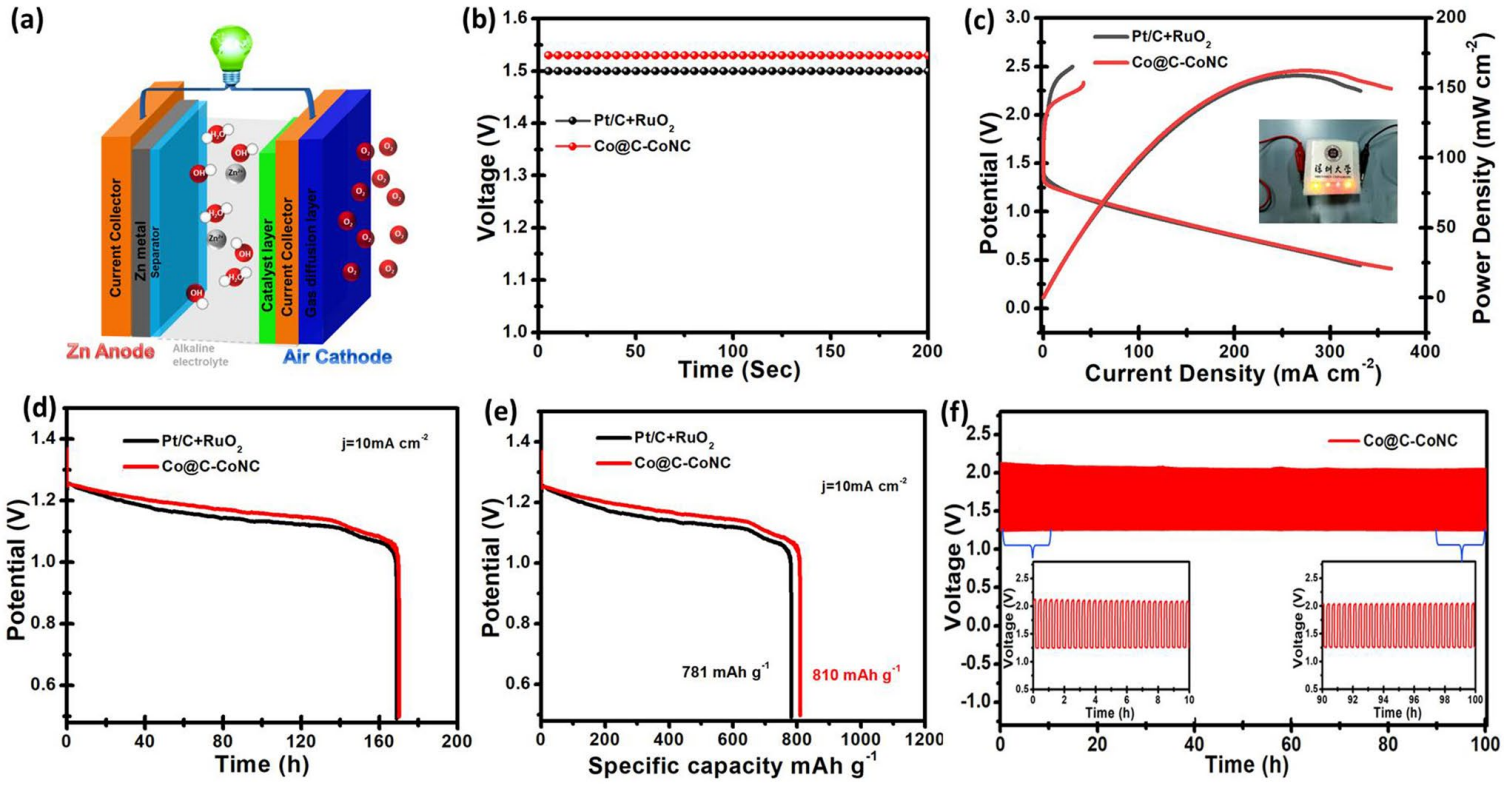
**a** Schematic of the ZAB; **b** Open circuit plots; **c** Charging and discharging polarization curves and the corresponding power densities for ZABs based on Co@C-CoNC and Pt/C + RuO_2_, in which the inset shows LEDs powered by a Co@C-CoNC based ZAB; **d** The galvanostatic discharge curves and **e** specific capacities of ZABs made by Co@C-CoNC and benchmark Pt/C + RuO_2_ catalysts at 10 mA cm^−2^, respectively; **f** Long-term cycling test at a current density of 2 mA cm^−2^ for Co@C-CoNC based ZABs

Encouraged by the high bifunctional electrocatalytic activity of Co@C-CoNC, the proof‐of‐concept test was performed to demonstrate the feasibility of Co@C-CoNC as an air cathode catalyst in a rechargeable ZAB (Fig. 6a). As a comparison, a ZAB was also constructed using commercial ORR catalyst Pt/C and OER catalyst RuO_2._ The open-circuit voltage of the Co@C-CoNC-based ZAB is about 1.53 V (Fig. 6b), which is higher than that based on Pt/C + RuO_2_ (1.50 V). The discharge/charge polarization curves and the corresponding power density plots are shown in Fig. 6c. At 1.0 V, Co@C-CoNC shows a current density of 97.7 mA cm^−2^, which is higher than that of Pt/C + RuO_2_ (86.5 mA cm^−2^). The Co@C-CoNC cathode also possesses a power density of 162.8 mW cm^−2^ (at 270.3 mA cm^−2^), while that based on the Pt/C + RuO_2_ cathode is only 158.9 mW cm^−2^ at 265.8 mA cm^−2^.

In addition, the ZAB based on Co@C-CoNC outperforms the Pt/C + RuO_2_ in the charge/discharge segments. Specifically, during the galvanostatic discharge at 10 mA cm^−2^, the Co@C-CoNC-based ZAB delivered a stable discharge voltage of around 1.2 V without any obvious degradation for a prolonged time period of ~ 140 h (Fig. 6d), implying the robustness of Co@C-CoNC cathode. Meanwhile, the specific capacity at 10.0 mA cm^−2^ is calculated to be as high as ~ 810 mAh g^−1^ normalized to the mass of consumed Zn (98.8% utilization of the theoretical capacity 820 mAh g^−1^), corresponding to an ultra-high energy density of 945 Wh kg^−1^ based on Zn (~ 87.0% of the theoretical energy density 1086 Wh kg^−1^), which are shown in Fig. 6e. Furthermore, the cycling stability of the Co@C-CoNC- based ZAB was measured by repeated discharging and charging (short cycle times, *i.e.,* 10 min per charge or discharge period) at 2 mA cm^−2^ for a prolonged time of ~ 100 h (retained a discharge- charge voltage gap of ~ 0.85 V in the whole course, Fig. 6f), signifying the foundation for the long-term cycling capacity of Co@C-CoNC catalyst. As an example of practical application, a Co@C-CoNC-based ZAB can light up a series of light-emitting diodes (Fig. 6c and Video S1). The above results indicate that the excellent bifunctional ORR/OER electrocatalytic activity of Co@C-CoNC can enable ZABs with high efficiency and long cycle life. Moreover, the excellent ZAB performance of the Co@C-CoNC catalyst is comparable or even superior to previously reported low-cost transition metal-based ZABs (Table S7).

### Density Functional Theory Calculations

**Fig. 7:**
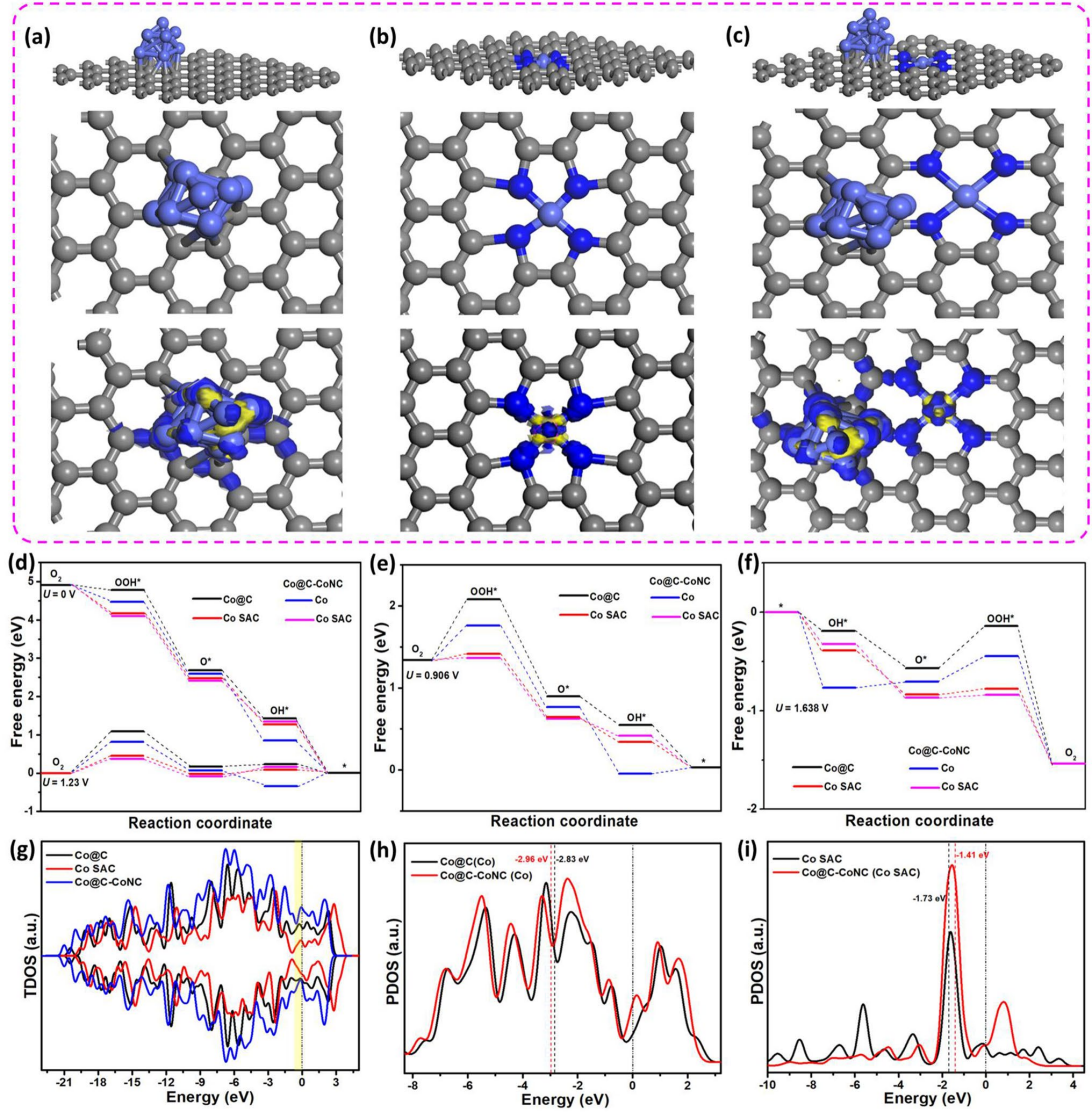
Atomic models with charge density difference plots for: **a** Co@C, **b** Co SAC (i.e., Co-N_4_), and **c** Co NP adjacent to Co SACs in Co@C-CoNC, in which the yellow and blue regions represent electron accumulation and depleted regions, respectively; **d-f** Free energy diagrams for ORR and OER, in which the black lines represent Co NP in bare Co@C, red lines represent Co SAC in Co-N_4_, and the blue and magenta lines represent the Co NP and the adjacent Co SACs in Co@C-CoNC; **g-i** Total and projected electron density of states (on d orbitals of Co) for bare Co@C, Co SAC and Co@C-CoNC catalysts

DFT calculations were performed to provide deeper insights into the intrinsic bifunctional electrocatalytic mechanism of our M–N-C catalysts. As shown in Figs. 7a–c and S32-S33, three ideal models representing metallic NPs (denoted as M@C), M–N_*x*_ SACs (denoted as M SAC), and the real sample systems of M–N–C (*i.e.,* isolated SACs with adjacent NPs) were constructed based on XANES and HAADF‐STEM results to identify the critical role of single atoms and adjacent NPs in facilitating the ORR/OER energetics. From the charge density difference plots, it is clearly observed that compared with metallic NPs and M–N_*x*_ site, coordinatively unsaturated NPs adjacent to the Co SAC (*i.e.,* Co–N_4_ configuration) conjointly change their charge-density electrons, resulting in an electron-rich state, which is highly favorable for the ORR/OER process by the rapid release of electron (Figs. 6a–c and S32-S33). Typically, both ORR and OER involve four elementary reaction steps, in which the ORR occurs via the creation of OOH* from adsorbed O_2_ and is further reduced to O* and OH*, whereas the OER proceeds in a reverse manner. The calculated OER/ORR free energy of metallic NPs, M–N_*x*_ SACs were added to compare with those of respective Co@C-CoNC, Fe@C-FeNC and Cu@C-CuNC at U = 0 and 1.23 V, as well as their practical half wave and low overpotentials on these metal sites were considered (Figs. 7d and S32-S33). Figure 7d shows that the ORR for metallic NPs, M–N_*x*_ SACs and M–N–C are downhill energy profiles at U = 0 V, representing the spontaneous exothermic ORR reactions with different reaction pathways and rate-determining steps (RDS). At the equilibrium potential of 1.23 V, the RDS on Co NP at Co@C and Co SAC show the hydrogenation of O_2_ molecule with the limiting barrier of 1.108 eV for Co NP at Co@C and 0.455 eV for Co SAC. Interestingly, this value further decreases to 0.782 and 0.388 eV for Co NP and Co SAC in Co@C-CoNC, revealing that the protonation of OH* becomes the RDS. Similarly, the limiting barrier of 1.154 eV for Fe NP at Fe@C and 0.395 eV for Fe SAC values further decrease to 0.675 and 0.339 eV for Fe NP at Fe@C and Fe SAC in Fe@C-FeNC (Fig. S32); and the limiting barrier of 0.96 eV for Cu NP at Cu@C and 0.734 eV for Cu SAC values further decrease to 0.695 and 0.670 eV for Cu NP and Cu SAC in Cu@C-CuNC (Fig. S33). These results emphasize the critical role of NPs adjacent to SAC sites in accelerating the ORR process. At the half-wave potential of Co@C-CoNC (0.906 V, Fig. 7e), Fe@C-FeNC (0.917 V, Fig. S32), and Cu@C-CuNC (0.829 V, Fig. S33), all the ORR reaction steps become downhill, again confirming the superior.

ORR activity of Co@C-CoNC and Fe@C-FeNC than Cu@CuNC, which is in good agreement with experimental ORR results and previous reports [44, 54–56]. Regarding OER, considering the real experimental conditions, the reaction energy barriers are 0.782 eV for Co NP and 0.460 eV for Co SAC in Co@C-CoNC, which are lower than that of the individual metallic Co (1.180 eV) and Co SAC (0.527 eV) (Fig. 7f). Similarly, the reaction energy barriers are 1.19 eV for Fe NP and 0.858 eV for Fe SAC in Fe@C-FeNC, which are lower than that of the metallic Fe (1.67 eV) and Fe SAC (0.877 eV) (Fig. S32); and these values are 1.704 eV for Cu NP and 1.68 eV for Cu SAC, again lower than that of the individual metallic Cu (1.97 eV) and Cu SAC (1.744 eV) (Fig. S33). In all, DFT calculations clearly revealed the enhanced electrocatalytic activity of Co@C-CoNC catalyst is accredited to both Co NP and Co SAC. These two types of metallic species conjointly reduce the reaction energy barriers and thus boost the ORR/OER kinetics through their fast adsorption/desorption ability of reaction intermediates.

To predict more precise information on the electronic effect of both NPs and adjacent SAC on Co@C-CoNC, the electronic density of states including total and projected density of states (DOS and PDOS) of three model structures, i.e. metallic NPs, SACs, and real samples of M–N–C were considered (Figs. 1g–i and S32-S33). When compared to bare metallic NPs and SAC models, M–N–C samples exhibited an increase in the electron-occupied state near the Fermi level, indicating that the coordinatively unsaturated NPs near the M–N_*x*_ configuration conjointly change the d-electrons density, and promote the carrier densities, which contribute to the enrichment of electron transfer ability between the surface of the catalyst and adsorbed reaction intermediates. Generally, the electrocatalytic reactions are highly dependent on the electronic coupling between the atomic orbitals of the adsorbates and the d-electron states of the active sites [44, 55, 57, 58]. Moreover, Figs. 7h, i and S32-S33 show the mutually self-regulated d-band centers of Co SAC (downshift to −1.41 eV) in the Co-N_4_ feature and Co NP (upshift towards −2.96 eV) in Co@C-CoNC, apparently facilitating optimal adsorption and desorption of reaction intermediates. Similarly, compared to bare NP and SAC counterparts, the d-band centers of Fe SAC downshift to −1.09 eV in the Fe-N_3_ feature, and Fe NP upshift towards −3.18 eV in Fe@C-FeNC and the d-band centers of Cu SAC downshift to −1.59 eV in Cu-N_5_ feature and Cu upshift towards −3.64 eV in Cu@C-CuNC. These results clearly suggest that the changes in the d-band center position of M–N_*x*_ SACs are closely related to the neighboring NP atom, which could induce the d-electron self-regulation property. According to Sabatier's principle and the d-band center theory, a more positive d-band center could result in stronger adsorption interaction between the active site and the adsorbates such as reaction intermediates and products [31, 56]. Following this rule, the downshift d-band center of SACs in the M–N–C with respect to SACs in bare M–N_*x*_ is in the order Fe@C-FeNC > Co@C-CoNC > Cu@C-CoNC, which is in good agreement with the experimental results for the ORR activity of the M–N–C samples (Fig. 4). Conversely, more negative d-band center suggests that the energy of antibonding states are lowered and easier to be filled. Hence, the interaction between adsorbates and catalyst surface could be weakened, which can result in reduced adsorption energy and meanwhile enhanced desorption ability for oxygen intermediates [31, 57–59]. It is worth noting that, the too-weakened desorption process could lead to difficult adsorption of the reaction products and will result in deprived electrocatalytic reaction kinetics. In this context, the highly active electrocatalyst is a material with a surface-oxygen interaction that is neither too strong nor too weak. Following this classical phenomenon, the negative shift of the d-band center of NPs in the M–N–C with respect to bare NPs in M@C is in the order Co@C-CoNC > Fe@C-FeNC > Cu@C-CoNC, which is consistent with the experimental results for OER activity of the M–N–C samples (Fig. 5). Consequently, our DFT predictions suggest that in an M–N–C system, moderate (i.e., balanced) d-band centers of SAC and adjacent NP is essential for realizing robust bifunctional electrocatalytic reaction, which could conjointly contribute/compensate appropriate binding interaction of oxygen intermediates to enrich bifunctional reaction kinetics. The electronic and geometric structures of SACs are highly sensitive to the local atomic coordination environment (i.e. their coordination feature and adjacent NP atoms), it could be a key factor to determine the functionality for either ORR, OER, or bifunctional ORR/OER catalysts. Notably, this d-band center tendency agrees very well with the previous reports [15, 31, 59]. This mechanistic study suggests that the moderately self-regulated d-band centers of Co SAC and its adjacent Co NP in a Co@C-CoNC are essential and conjointly they provide/compensate appropriate binding interaction of oxygen intermediates and thus result in lower free energies to possess enhanced bifunctional ORR and OER activity. Hence, we believe that the co-existence of an adequate amount of metal-containing NPs and high content of M − N_*x*_ active sites are indispensable for the superior bifunctional electrocatalytic ORR/OER performance.

## Conclusions

In summary, we demonstrated an innovative way for the fabrication of a new class of metal-impregnated CD-based MOFs strategy to synthesize M–N–C catalysts consisting of both metal nanoparticles and isolated single-atom sites (M/M_*x*_C). Among the transition metals used, the Fe@C-FeNC and Co@C-CoNC exhibit better ORR activity than Cu@C-CuNC and the commercial Pt/C. Furthermore, as a bifunctional catalyst, the Co@C-CoNC exhibits the best performance, showing a Δ*E* of ~ 0.732 which is much lower than that of Fe@C-FeNC and Cu@C-CuNC, as well as most of the robust bifunctional catalysts reported to date. By taking all experimental results and DFT calculations into account, it is signifying the strong electronic correlation between metallic Co NPs sites and the adjacent Co-N_4_ SAC sites in a Co@C-CoNC catalyst can increase the d-electron density near the Fermi level and thus effectively optimize the adsorption/desorption of intermediates in ORR/OER, which bestow the superior electrocatalytic performance. We believe that the proposed strategy of rational and controlled design of CD-MOF materials and their derivatives could benefit the manufacturing of non-precious transition metals-based electrocatalysts, which may shed light on the further development of catalysts as well as other eminent research fields.

### Supplementary Information

Below is the link to the electronic supplementary material.

Supplementary file1 (MP4 2832 kb)

Supplementary file2 (PDF 4583 kb)
