# Supplementary material for: Mutual Self-Regulation of d-Electrons of Single Atoms and Adjacent Nanoparticles for Bifunctional Oxygen Electrocatalysis and Rechargeable Zinc-Air Batteries
Source: Nanomicro Lett. 2023 Feb 11;15:48. doi: 10.1007/s40820-023-01022-8 (PMC9922344; doi:10.1007/s40820-023-01022-8)
Supplement: Supplementary file 2 — Supplementary file2 (PDF 4583 kb) [file 40820_2023_1022_MOESM2_ESM.pdf]

Supporting Information for

Mutual Self-Regulation of d-Electrons of Single-Atoms and Adjacent Nanoparticles for Bifunctional Oxygen Electrocatalysis and Rechargeable Zinc-Air Batteries

Sundaram Chandrasekaran[1, 2, †, *], Rong Hu[1, †], Lei Yao[3], Lijun Sui[4], Yongping Liu[2], Amor Abdelkader[5], Yongliang Li[1], Xiangzhong Ren[1] and Libo Deng[1, *]

[1]College of Chemistry and Environmental Engineering, Shenzhen University, Shenzhen 518060, P. R. China

[2]College of Chemistry and Bioengineering, Guilin University of Technology, Guilin 541004 P. R. China

[3]Shenzhen Key Laboratory of Special Functional Materials, Shenzhen Engineering Laboratory for Advanced Technology of Ceramics, Guangdong Research Center for Interfacial Engineering of Functional Materials, College of Materials Science and Engineering, Shenzhen University, Shenzhen 518060, P. R. China

[4]Shanghai Key Laboratory for R&D and Application of Metallic Functional Materials, Institute of New Energy for Vehicles, School of Materials Science and Engineering, Tongji University, Shanghai 201804, P. R. China

[5]Department of Design and Engineering, Faculty of Science & Technology, Bournemouth University, Poole, Dorset BH12 5BB, United Kingdom

†Sundaram Chandrasekaran and Rong Hu contributed equally to this work.

*Corresponding authors. E-mail: Denglb@szu.edu.cn (Libo Deng), yes.chandrasekaran@gmail.com or chandru@glut.edu.cn (Sundaram Chandrasekaran)

S1 Material Characterizations

The crystalline structure and phase purity were assessed using PXRD (XRD, Bruker/D8 Advance with Cu Kα radiation ($\lambda = 1.5406$ Å)). The surface morphology, nano-, and micro-structures of the prepared materials were identified by Field-Emission Scanning Electron Microscopy (FE-SEM, JEOL JSM-7800F), Transmission Electron Microscopy (TEM, JEM-2100) and Aberration corrected high-resolution Scanning Transmission Electron Microscope (AC-STEM, FEI TALOS F200X). TGA was carried out using TGA/DSC 3+; Mettler Toledo. The N_2 adsorption−desorption isotherms were obtained at 77 K using a specific surface area analyzer of Micromeritics ASAP 2460. The metal concentrations of the prepared samples were measured by ICP-OES (SPECTRO ARCOS ICP-OES analyzer; Arcos II MV). XPS was conducted on a Thermo Scientific™ ESCALAB™ 250Xi spectrometer. Raman spectra were acquired on a Renishaw INVIA REFLEX Raman spectrometer with a 532 nm laser source. The X-ray absorption fine structure spectra (XANES) Fe, Co, and Cu K-edge were collected at the BL07A1 beam-line of the National Synchrotron Radiation Research Center (NSRRC). The data were collected in fluorescence mode using a Lytle detector while the corresponding reference samples were collected in transmission mode. The samples were ground and uniformly daubed on the special adhesive tape. The obtained XAFS data was processed in Athena (version 0.9.26) for background, pre-edge line, and post-edge line calibrations. Then Fourier transformed fitting

was carried out in Artemis (version 0.9.26). The k^3 weighting, k-range of 3–12 $Å^{-1}$, and R range of 1–3 Å were used for the fitting. The four parameters, coordination number, bond length, Debye-Waller factor, and E_0 shift (CN, R, σ^2, ΔE_0) were fitted without anyone being fixed, constrained, or correlated. For Wavelet Transform analysis, the $\chi(k)$ exported from Athena was imported into the Hama Fortran code. The parameters were listed as follows: R range was about ~ 1–4 Å, k range was about ~ 0–12 $Å^{-1}$; k weight was 2; and Morlet function with $\kappa = 10$, $\sigma = 1$ was used as the mother wavelet to provide the overall distribution.

S2 Growth and Analysis of Single Crystals

The preparation of a mother solution for all CD-MOFs followed the general procedure of dissolving the γ-CD (520 mg) and the alkali metal salt (NaOH, 65 mg) in 10 mL H_2O and stirring for 45 min, then filtering the solution using a sterile syringe filter with a 0.45 µm pore sized hydrophilic Polyethersulfone (PES) membrane, and subsequently allowing slow vapor diffusion of MeOH into the filtrated solution to grow crystals for seven days. The grown crystals were collected, washed with methanol, and allowed to dry in the air. Single crystals of Co-CD-MOF, Fe-CD-MOF, and Cu-CD-MOF were prepared by adding a 12 mM solution of cobalt hydroxide, iron hydroxide, and copper hydroxide solution, respectively, into the mother solution under the same conditions as CD-MOF.

The single-crystal structure was analyzed using an X-ray single-crystal diffraction instrument, 'Bruker APEX-II CCD' diffractometer with Cu Kα. The crystal was kept at the setting room temperature during data collection. Using $Olex^2$ [S1], the structure was solved with the ShelXT [S2] structure solution program using intrinsic phasing and refined with the ShelXL [S3] refinement package using Least Squares (LS) minimization.

S3 Preparation of Catalysts

Due to the large scale of the catalyst, complete grinding was treated before the electrocatalytic test. Initially, 4 mg of the as-prepared catalyst and 50 µL Nafion solution (5 wt.%) were dispersed into 950 µL ethanol and sonicated for 30–45 min to form a homogenous catalyst ink. Further, to prepare the working electrode, a 10 µL aliquot of the homogeneously prepared catalyst ink (~0.239 mg cm^{-2}) was drop casted on the surface of the pre-cleaned Rotating Disk Electrode (RDE) or Rotating Ring Disk Electrode (RRDE) (diameter of 4 mm). Finally, the modified working electrode was dried to perform the electrochemical measurements in 0.1 M KOH and/or 0.1 M $HClO_4$ electrolytes, where the mass loading of the commercial 20 wt.% Pt/C was ~0.12 mg cm^{-2}.

S4 Electrochemical Measurements

All of the electrochemical tests were conducted on a CHI 760E electrochemical instrumentation equipped with a high-speed rotator (RRDE-3A; Rotating Ring Disk Electrode). The typical three-electrode cell configuration consisted of Ag/AgCl (saturated KCl solution), Pt wire, and RDE or RRDE as a reference, counter, and working electrodes, respectively. All electrochemical measurements were performed in 0.1 M KOH or 0.1 M $HClO_4$ solution purged with either oxygen (O_2) or nitrogen (N_2) for at least 30 min before measurement at room temperature. Finally, the potential, measured against the Ag/AgCl electrode, was converted to a potential (V) *vs.* RHE (reversible hydrogen electrode) according to the Nernst equation [S4, S5]:

$$E_{RHE} = E_{Ag/AgCl} + 0.197 + 0.0591 * pH \quad (S1)$$

CV curves were recorded in oxygen (O_2) or Nitrogen (N_2) saturated 0.1 M KOH electrolyte solution several times at a scan rate of 10 mV s^{-1} until reproducible curves were obtained. For the OER test, the polarization curves were obtained at a rotating rate of 1600 rpm with a scan rate of 10 mV s^{-1}, corrected by iR-compensation. For ORR, the linear sweep voltammetry (LSV) measurements were performed in an O_2-saturated 0.1 M KOH solution at a scan rate of 10 mV s^{-1} with the rotation speed varying from 400–2400 rpm. Based on LSV polarisation measurements, the electron transfer number (n) and kinetic current density (J_k) were examined by employing the Koutecky-Levich (K-L) Eq. (2) [S6]:

$$\frac{1}{J} = \frac{1}{B\omega^{1/2}} + \frac{1}{J_K} \quad \text{(S2)}$$

where,

$$\mathrm{B} = 0.62\mathrm{nF}C_0(D_0)^{2/3}v^{-1/6} \quad \text{(S3)}$$

$$J_K = nFkC_0 \quad \text{(S4)}$$

where J represents the measured current density, J_K represents the kinetic limiting current densities, ω denotes the angular velocity of the disk ($\omega = 2\pi N$ where N is the linear rotation speed), and n signifies the overall number of electrons transferred in oxygen reduction. F is the Faraday constant (F = 96485 C mol^{-1}), C_0 is the bulk concentration of O_2, v is the kinematic viscosity of the electrolyte, k is the electron transfer rate constant, and D_0 is the diffusion coefficient of O_2 in the electrolyte [S7].

The electron transfer number (n) and the corresponding peroxide yield (HO_2^- %in alkaline solution and H_2O_2 % in acidic solution) were determined based on the disk and ring currents using the following equations [S7]:

$$\mathrm{n} = 4 \times \frac{I_{disk}}{I_{dsk}+I_{ring}/N} \quad \text{(S5)}$$

$$peroxide\ \% = 100\times\frac{2I_{ring}/N}{I_{disk}+I_{ring}/N} \quad \text{(S6)}$$

where, I_{disk} and I_{ring} are the faradic disk and ring currents, respectively. N is the collection efficiency of the ring electrode, which is determined to be ~0.43. For the ORR and OER Tafel plots, the kinetic currents were considered after the mass-transport correction of RDE data based on previously reported literature [S8, S9].

S5 Assembly and Testing of zinc (Zn) Metal-Air Battery (ZAB)

The catalyst was used as an air cathode on the electrode composite substrate (which is superimposed and compacted by Ni foam, hydrophobic breathable membrane, and carbon paper in sequence from bottom to top). The polished zinc (Zn) plate was used as an anode and the electrolyte is 6 M KOH. The battery device was assembled by the sequence of air cathode and Zn plate anode, and the circulation pump device was connected to the inlet and outlet of the outer side of the battery mold. The circulating flow rate of the circulating pump was set at 10 mL min^{-1} to form a self-made rechargeable zinc-air battery (ZAB) device with a circulating electrolyte. To prepare an air cathode, catalyst, conductive carbon black, and polytetrafluoroethylene (PTFE) dispersion were added to 1 mL of ethanol in the ratio of 3:1:3 and sonicated for 30 min to get a uniformly dispersed catalyst slurry [S10]. Finally, the slurry was evenly coated on the electrode composite matrix, the mass load of the catalyst is 2 mg cm^-

[2] and then dried in an oven at 65 ℃ for 12 h for standby. For comparison, Pt/C+RuO_2 based ZAB was prepared with Pt/C+RuO_2 catalyst (1 mg cm^{-2}) with the mass ratio of 1:1. The open-circuit voltage and polarization curve of the rechargeable zinc-air battery was tested in CHI 760E electrochemical workstation OCP (open circuit potential) mode and LSV mode. The assembled rechargeable ZAB chamber was filled with 6 M KOH solution through the circulating pump and left to stand for 5 min. The test was carried out after the electrolyte is completely soaked. During the polarization tests, the scan rate of 5 mV s^{-1} was maintained. The cycling stability of the ZAB was tested by repeated discharging and charging for 10 min for each segment, at a constant current of 2 mA cm^{-2}.

S6 Density Functional Theory Calculations

All DFT calculations are performed with the CASTEP module in Materials Studio [S11, S12]. within the Perdew–Burke–Ernzerhof (PBE) generalized gradient approximation (GGA) was used for the DFT exchange-correlation energy, and a 550 eV kinetic energy cutoff was assigned to the plane-wave basis set, and the ultra-soft pseudopotentials were used. A gamma-centered 5×5×5 k-mesh was used to optimize the structure until all residual forces decline below 0.02 eV $Å^{-1}$, while a 5×5×1 k-mesh was adopted for electronic structure calculations. For free energy calculations, the energy cutoff for plane wave expansions was set to 550 eV, and the spin-polarized PBE exchange-correlation function, including the semi-empirical DFT-D correction of the Grimme method that was applied for dispersion corrections, while the energy and forces are converged to $1×10^{-5}$ eV and 0.02 eV/Å, respectively. A vacuum space of 15 Å was applied. The self-consistent field (SCF) tolerance was $1×10^{-5}$ eV, and the Brillouin zone was sampled using a 4×4×1 k-mesh using the Monkhorst-Pack scheme. The geometry is optimized until all residual forces are less than 0.01 eV $Å^{-1}$. Initially, the models for DFT calculations are constructed based on the characterization, analysis, and approximation of our prepared catalysts. As for Co@C-CoNC, the Co-N_4 SAC site was used as the structural model based on the EXAFS fitting result. Additionally, to have a good comparison, we choose three layers of Co atoms (9 atoms) on the carbon surface to construct Co@C three-layer model. Similar models were used for all other catalysts such as Fe@C-FeNC and Cu@C-CuNC.

At first, we calculated the intermediates adsorption energies of ORR or OER intermediates by the following approach based on previous reports [S13-S15],

$$\Delta E_{*O} = E(*O) - E(*) - (E_{H_2O} - E_{H_2}) \tag{S7}$$

$$\Delta E_{*OH} = E(*OH) - E(*) - (E_{H_2O} - \frac{1}{2}E_{H_2}) \tag{S8}$$

$$\Delta E_{*OOH} = E(*OOH) - E(*) - (2E_{H_2O} - \frac{3}{2}E_{H_2}) \tag{S9}$$

For the conversion of the adsorption energy at zero temperature into the Gibbs free energy at the ambient temperature and pressure, the entropy (ΔS) and zero-point energy corrections (ΔZPE) to the adsorption energy together with the solvation correction were considered. Hence, a solvation correction energy of -0.22 eV was used for ΔE_{*OOH} and ΔE_{*OH}, since molecules of H_2O can solvate *OOH and *OH moieties with hydrogen bond, while the hydrogen bond is absent for *O [S13]. Therefore, the adsorption Gibbs free energies for each reaction can be expressed as follows:

$$\Delta G_{*O} = \Delta E_{*O} + (\Delta ZPE - T\Delta S) \text{ (eV)} \tag{S10}$$

$$\Delta G_{*OH} = \Delta E_{*OH} + (\Delta ZPE - T\Delta S) - 0.22 \text{ (eV)} \tag{S11}$$

$$\Delta G_{*OOH} = \Delta E_{*OOH} + (\Delta ZPE - T\Delta S) - 0.22\ (eV) \quad (S12)$$

The alkaline ORR reaction kinetics is generally accepted by the following process [S15, S16]:

$$O_2(g) + * \rightarrow O_2* \quad (S13)$$

$$O_2* + H_2O(l) + e^- \rightarrow OOH* + OH^- \quad (S14)$$

$$OOH* + e^- \rightarrow O* + OH^- \quad (S15)$$

$$O* + H_2O(l) + e^- \rightarrow *OH + OH^- \quad (S16)$$

$$OH* + e^- \rightarrow OH^- + * \quad (S17)$$

Typically, a robust electrocatalyst should be capable to expedite ORR above the equilibrium potential, however, it necessitates all four electron charge-transfer steps to have reaction free energy of a similar magnitude at zero potential (i.e., 4.92 eV/4 = 1.23 eV) [S15]. This is equal to all the reaction free energy being zero at the equilibrium potential, 1.23 V. So, the reaction free energy of equations (S14-17) for the ORR can be calculated from the following equations [S15]:

$$\Delta G_1 = \Delta G_{*OOH} - 4 \times 1.23\ (eV) \quad (S18)$$

$$\Delta G_2 = \Delta G_{*O} + \Delta G_{*OOH} \quad (S19)$$

$$\Delta G_3 = \Delta G_{*OH} + \Delta G_{*O} \quad (S20)$$

$$\Delta G_4 = -\Delta G_{*OH} \quad (S21)$$

The rate-determining step (RDS) is the elementary reaction with minimum reaction free energy that can be obtained by,

$$\eta_{ORR} = min\{\Delta G1, \Delta G2, \Delta G3, \Delta G4\} - 1.23\ eV \quad (S22)$$

Supplementary Figures and Tables

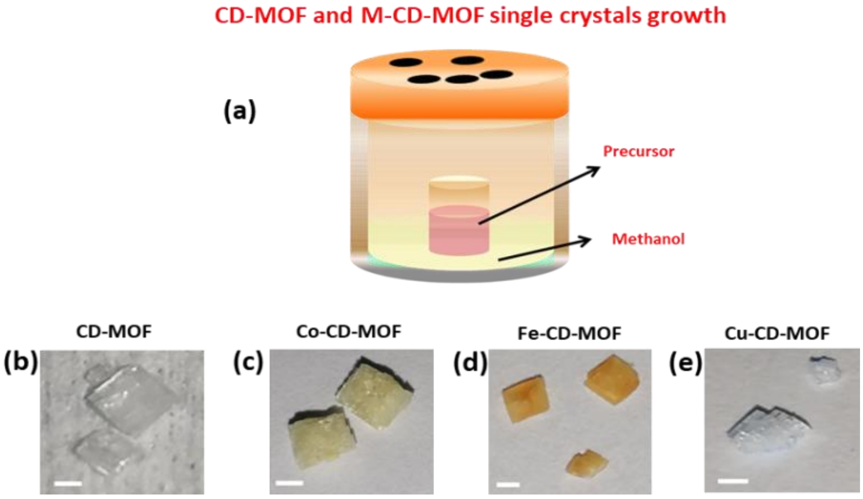

Scheme S1 a Schematic of single crystals growth via vapor diffusion method; **b-e** Photographs of the CD-MOF crystals (scale bars ~ 1mm, the size of the crystals were around ~2.5–3.0 mm)

Single crystal data:

Bare CD-MOF: Structure is Triclinic, $C_{96}H_{164}Na_2O_{82}$

Lattice parameters for CD-MOF is a=15.1513(5) b=16.8018(5) c=17.0695(6),

Unit-cell volume = 4262.9 $Å^3$ and Space group: P1.

α=92.009(2) β=97.335(2) γ=97.887(2);

Notably, numerous attempts were made to identify the single crystal XRD results/test for all the metal-impregnated MOFs (i.e. M-CD-MOF, M = Co, Fe, or Cu). However, all the M-CD-MOF crystals revealed weak diffraction patterns. Thus it was hard to solve the crystal structure.

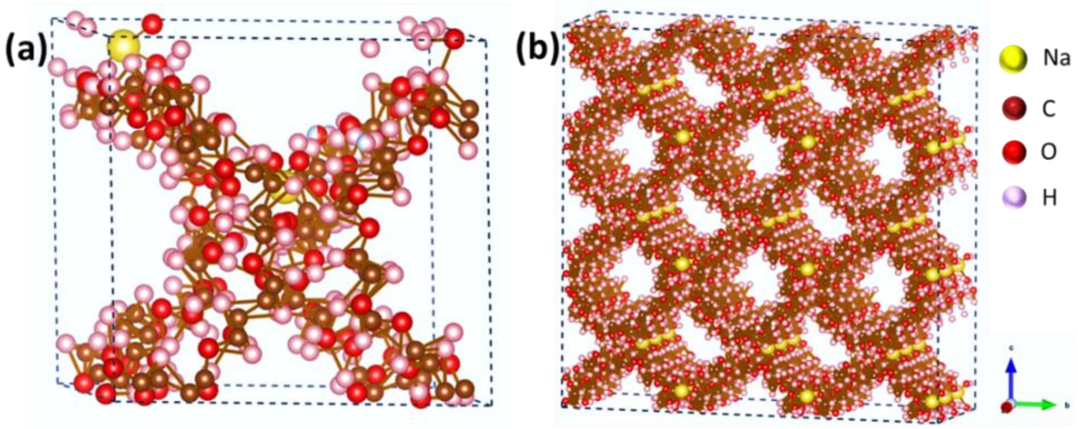

Fig. S1 Crystal structure of the bare CD-MOF derived from single-crystal XRD analysis

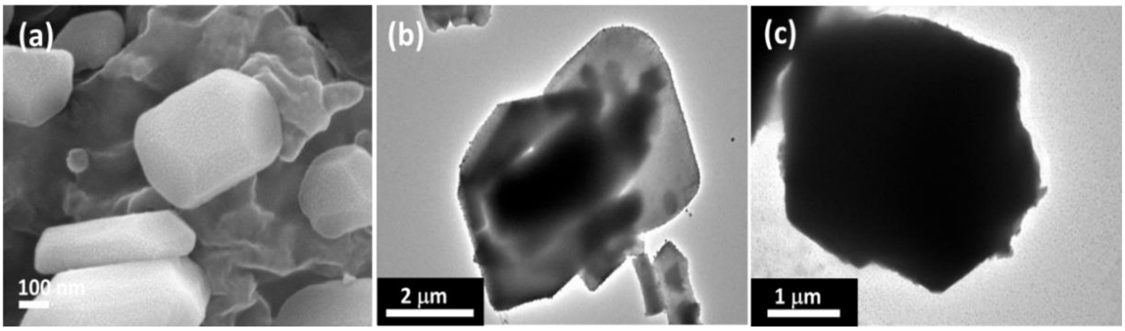

Fig. S2 a-c FE-SEM and TEM images of Fe-CD-MOF

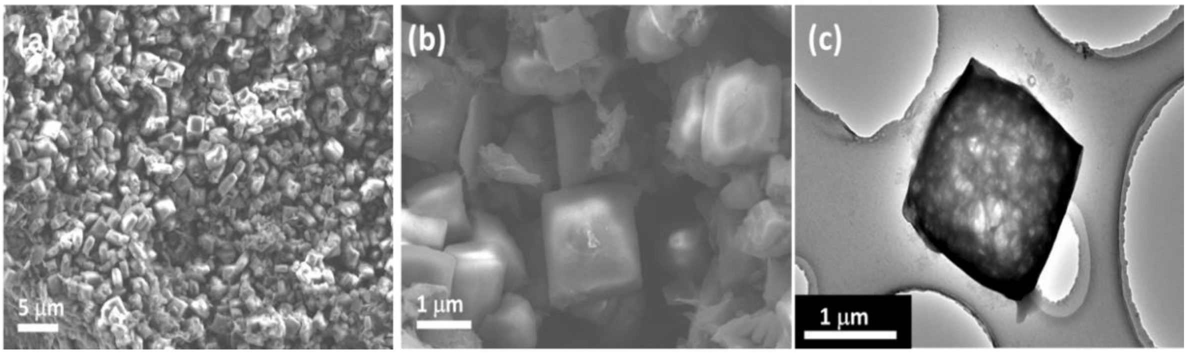

Fig. S3 a-c FE-SEM and TEM images of Cu-CD-MOF

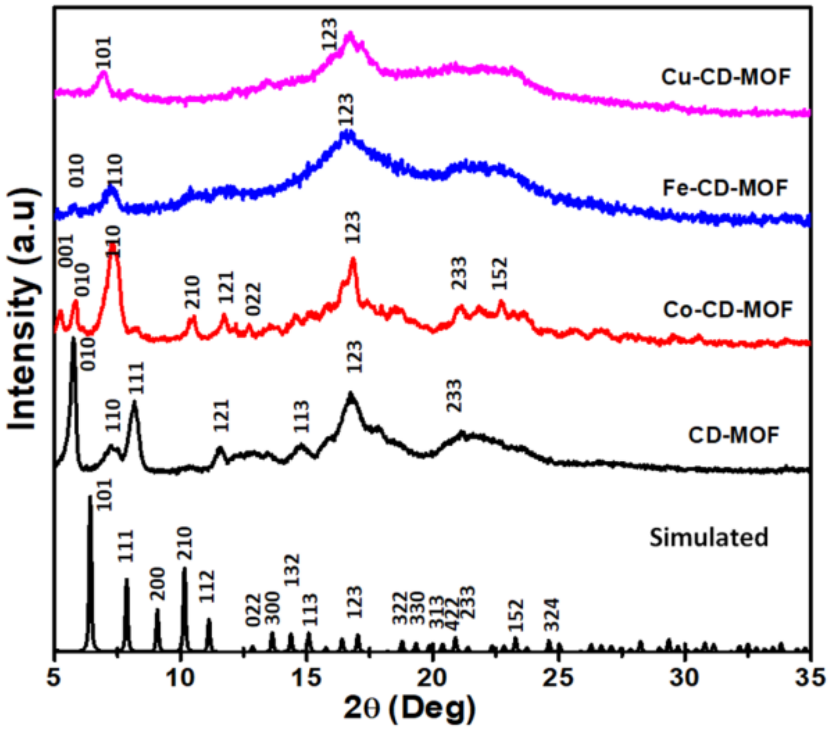

Fig. S4 XRD patterns of the CD-based MOFs and simulated pattern for CD

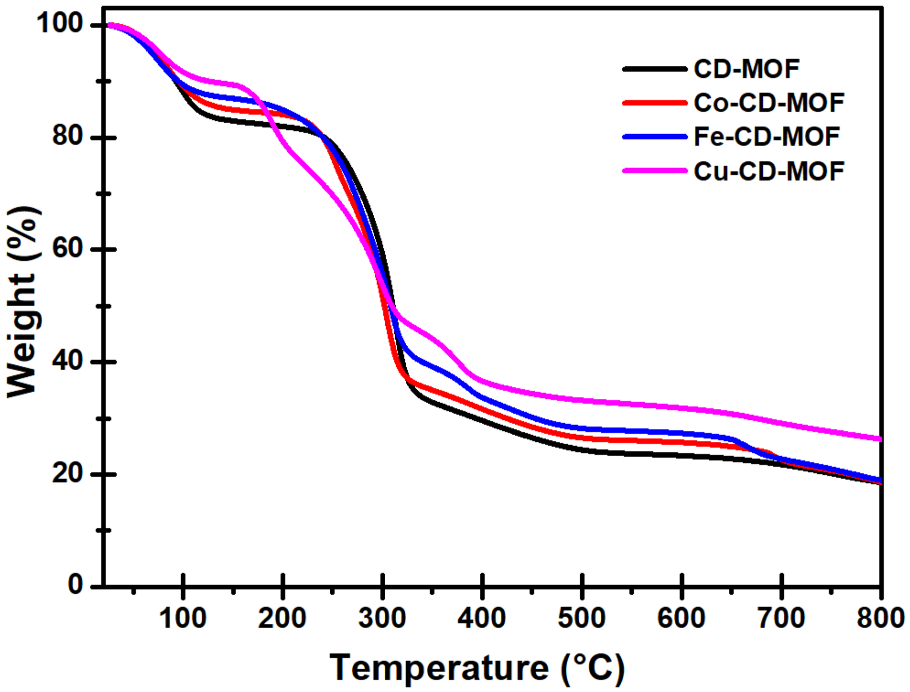

Fig S5 TGA profile of the as-prepared CD-based MOFs

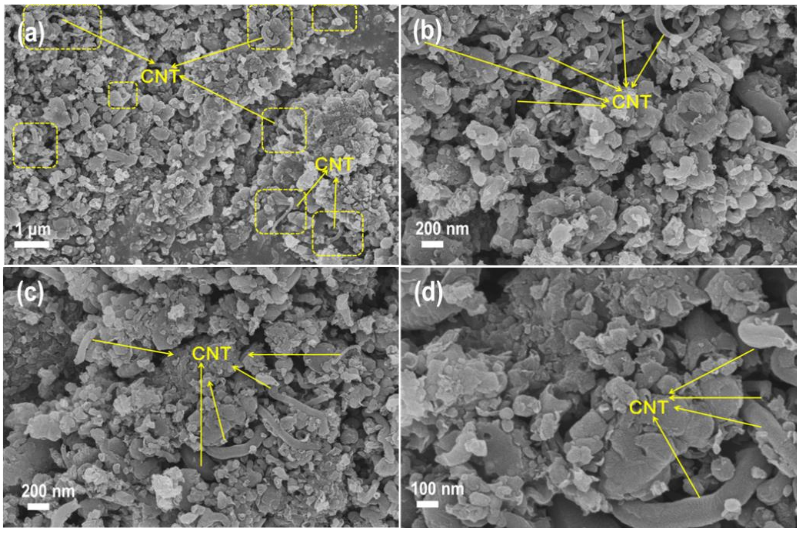

Fig. S6 a-d FE-SEM images of Co@C-CoNC catalyst

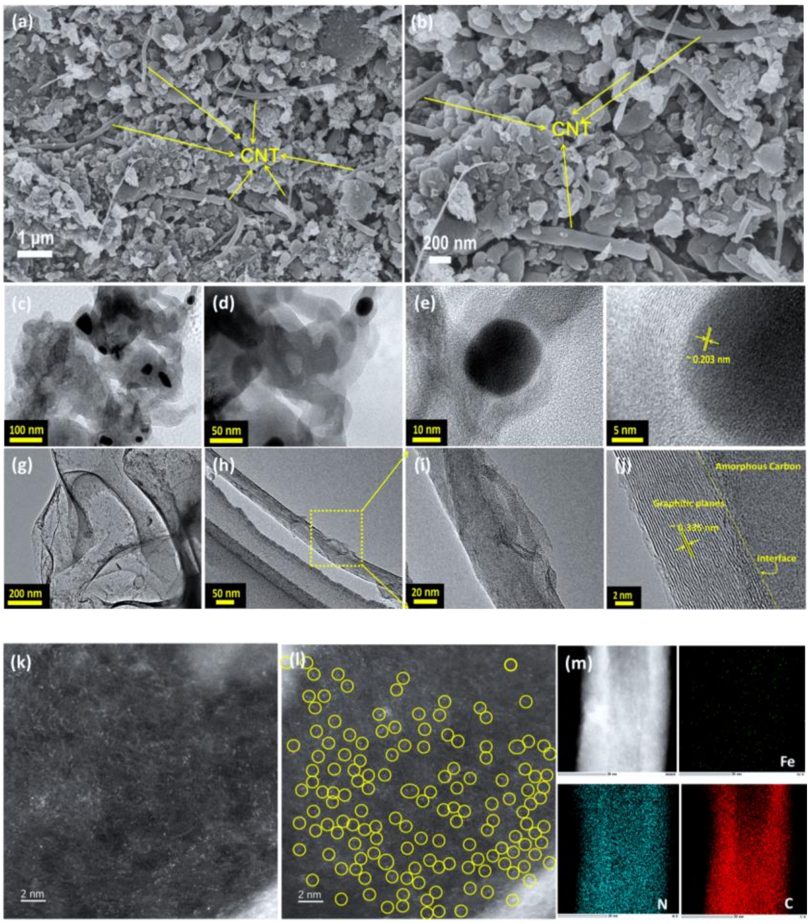

Fig. S7 a-b FE-SEM, **c-j** TEM, and HR-TEM, **k-l** HAADF-STEM images of Fe@C-FeNC catalyst at different areas with different magnifications. Microscopic images indicate the co-existence of carbonaceous hybrids of porous graphitic carbon and carbon nanotubes, with small-sized Fe/Fe_3C nanoparticles in **a-i**, **j** showing the existence of graphitic and amorphous phases in Fe@C-FeNC catalyst. The STEM images **k-l** show the isolated Fe-SACs on the hybrid carbonaceous matrix. **m** EDS mapping of Fe@C-FeNC catalyst

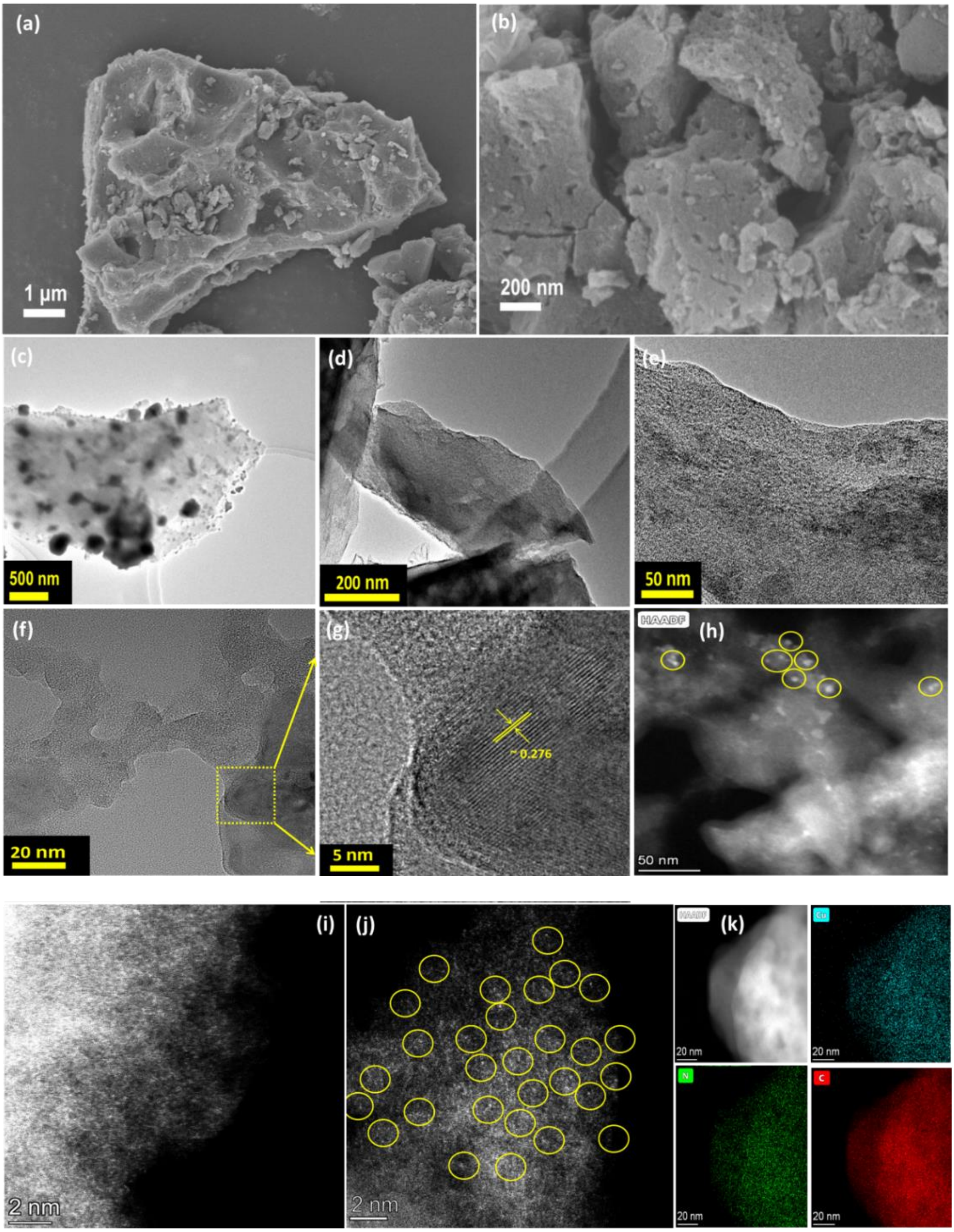

Fig. S8 a-b FE-SEM, **c-g** TEM and HR-TEM, **h-j** HAADF-STEM images of Cu@C-CuNC catalyst. Microscopic images indicate the co-existence of carbonaceous hybrids of porous graphitic carbon and small-sized Cu/CuC_8 nanoparticles in **a-h**. The STEM images **i-j** show the isolated Cu-SACs on the N-C matrix. **k** EDS mapping of Cu@C-CuNC catalyst

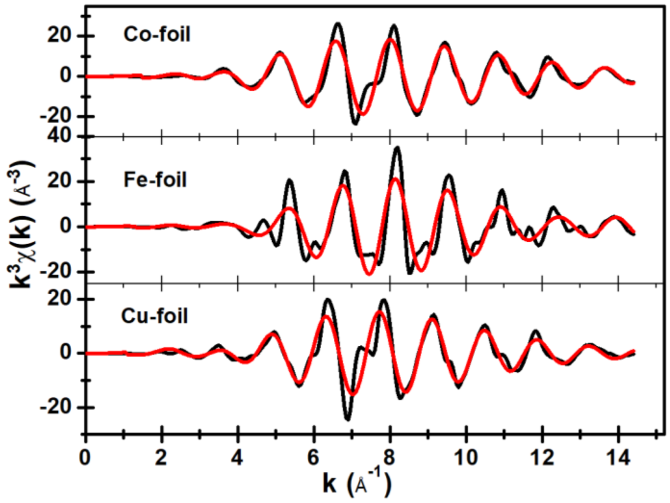

Fig. S9 EXAFS *k* space fitting curves of reference metal foils

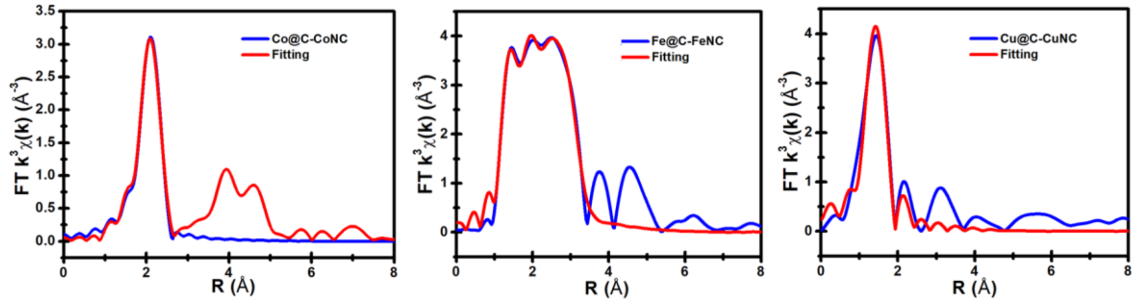

Fig. S10 EXAFS *R* space fitting curves of Co@C-CoNC, Fe@C-FeNC, and Cu@C-CuNC catalysts

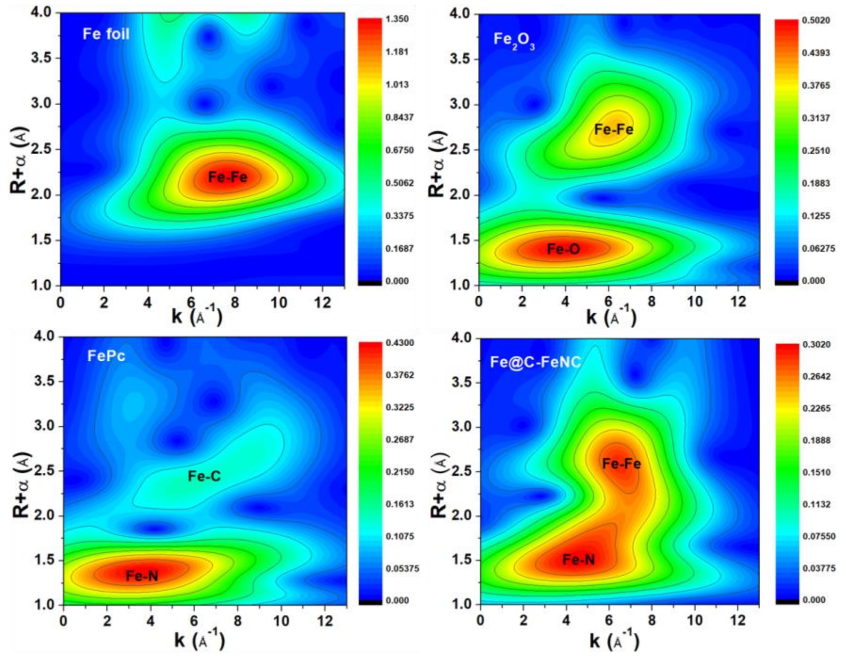

Fig. S11 EXAFS wavelet transforms for Fe@C-FeNC catalyst and its corresponding reference samples

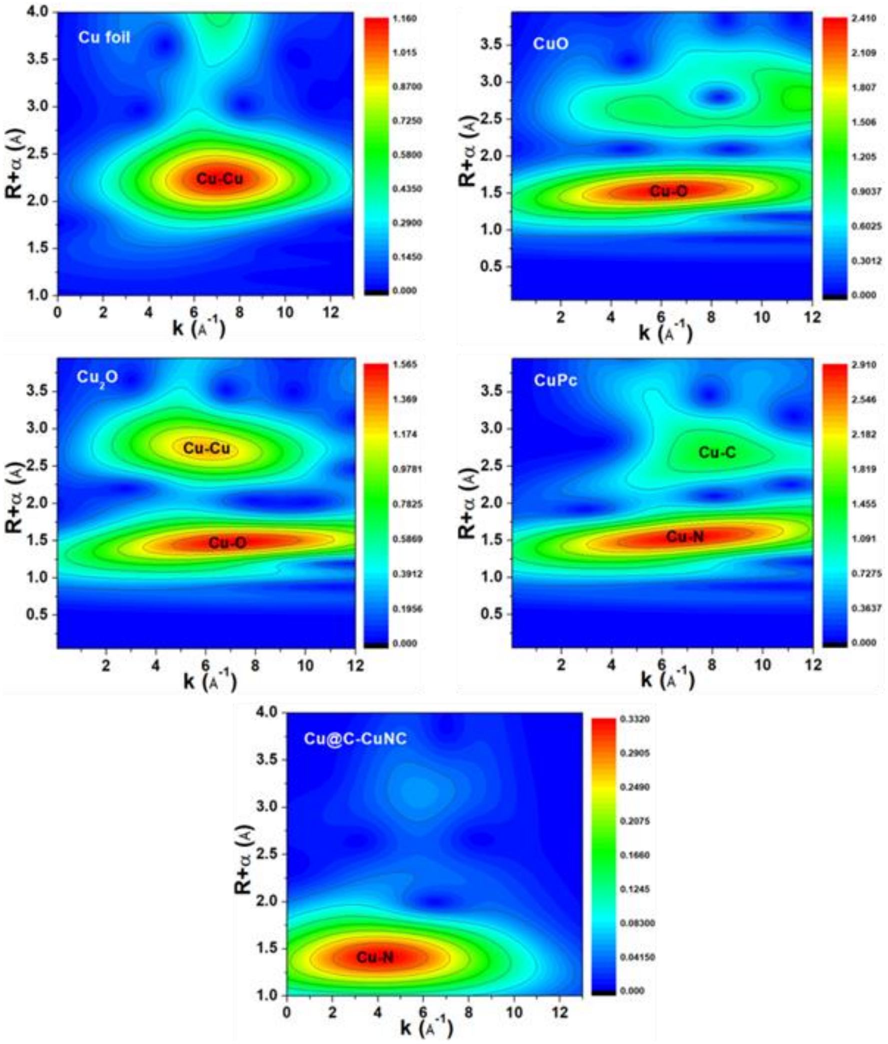

Fig. S12 EXAFS wavelet transforms for Cu@C-CuNC catalyst and its corresponding reference samples

Table S1 EXAFS fitting parameters at the Fe, Cu, Co K-edge for various samples（S_0^2=0.70, 0.86, 0.74）

**Sample**	**Shell**	***N***^{a}	***R*(Å)**^{b}	σ^2**×10**^{3}**(Å**^{2}**)**^{c}	**Δ*E*$_0$ (eV)**^{d}	***R* factor**
Co@C-CoNC	Co-N	4.0±1.3	1.91±0.01	3.4±0.1	-4.8±0.3	0.005
	Co-Co	8.0±0.2	2.50±0.01	5.4±0.6	8.5±1.1	
Fe foil	Fe-Fe	8*	2.47±0.01	4.8±0.9	6.6±1.3	0.002
	Fe-Fe	6*	2.85±0.01	6.1±1.7	5.2±2.6	
Fe@C-FeNC	Fe-N	3.0±0.5	1.95±0.01	4.9±1.5	-4.4±2.4	0.002
	Fe-Fe	4.3±0.8	2.51±0.01	10.3±1.7	-14.9±1.2	
	Fe-Fe	5.6±0.9	3.37±0.01	13.6±1.5		
Cu foil	Cu-Cu	12*	2.54±0.01	8.9±0.3	4.5±0.5	0.002
Cu@C-CuNC	Cu-N	5.4±0.9	1.97±0.01	10.0±2.1	-2.2±1.7	0.011

$^a N$: coordination numbers; $^b R$: bond distance; $^c \sigma^2$: Debye-Waller factors; ^{d} ΔE_0: the inner potential correction. *R* factor: goodness of fit.

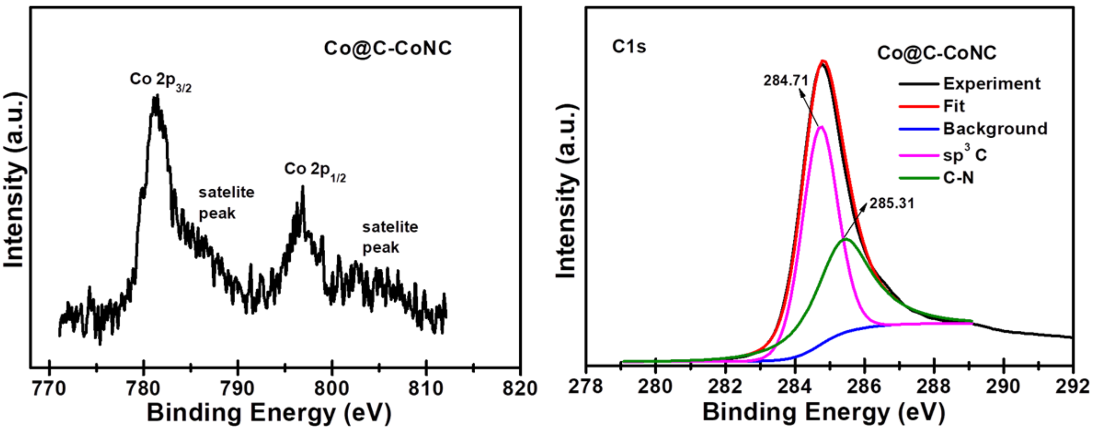

Fig. S13 The core-level XPS spectra of Co2p, and C1s for the Co@C-CoNC catalyst

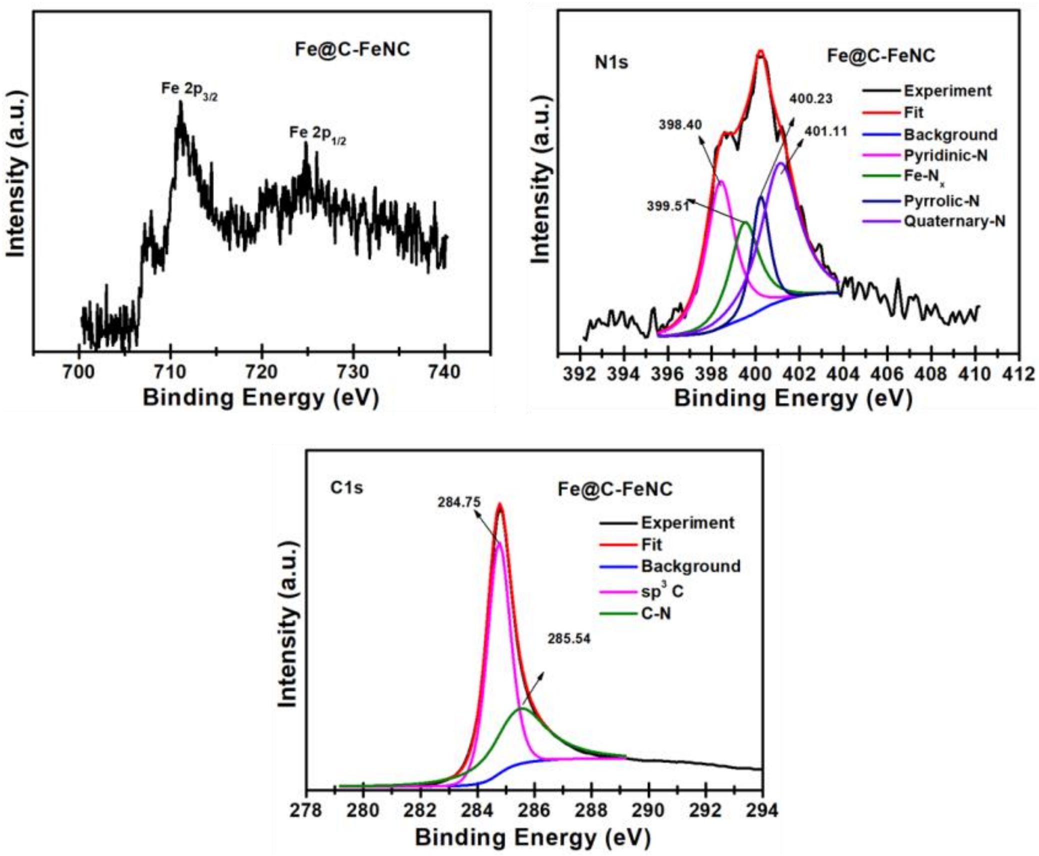

Fig. S14 The core-level XPS spectra of Fe2p, N1s, and C1s for the Fe@C-FeNC catalyst

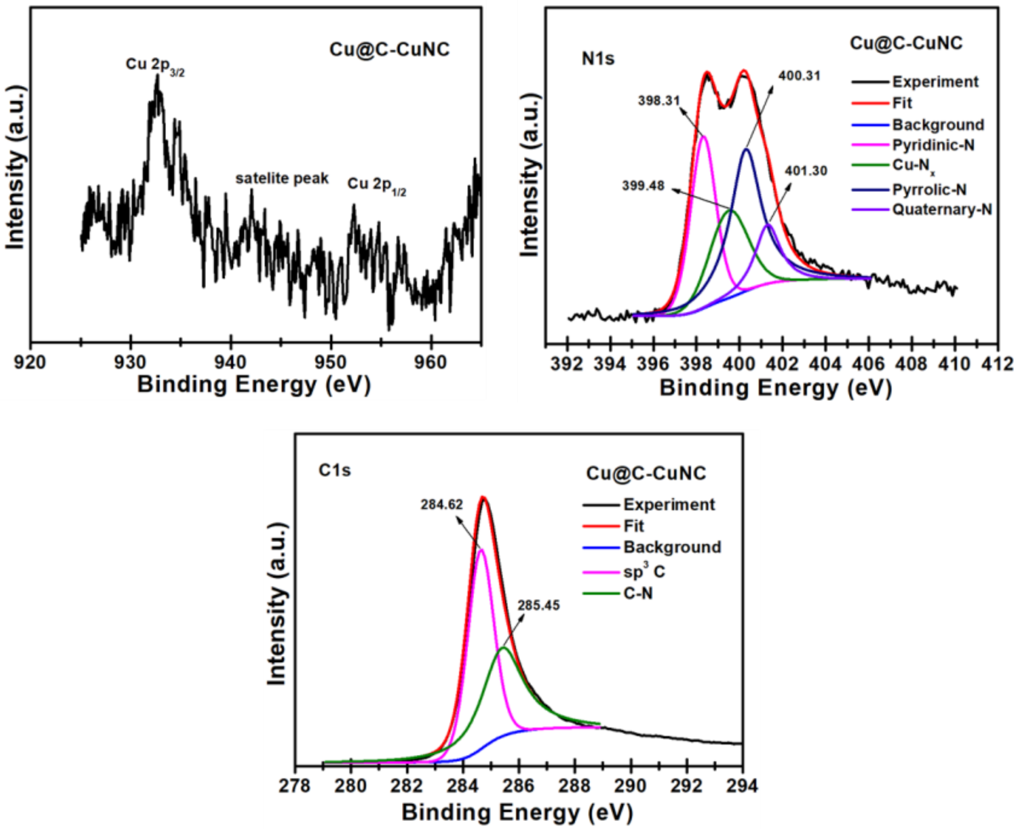

Fig. S15 The core-level XPS spectra of Cu2p, N1s, and C1s for the Cu@C-CuNC catalyst

Table S2 The chemical compositions of the prepared catalysts as determined by XPS

Catalysts	Carbon (wt%)	Metal (wt%)	Nitrogen (wt%)
Co@C-CoNC	92.38	2.44	5.18
Fe@C-FeNC	93.51	2.76	3.73
Cu@C-CuNC	92.72	1.45	5.83

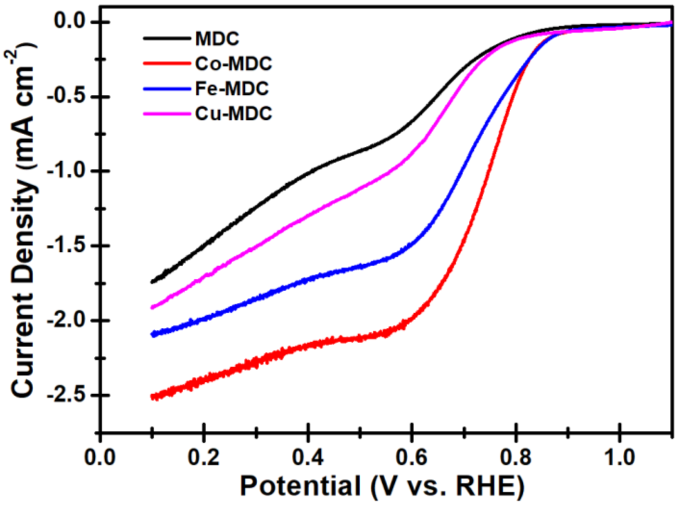

Fig. S16 ORR polarization curves for the bare MDC and M-MDC (M= Co, Fe and Cu) samples in 0.1 M KOH electrolyte

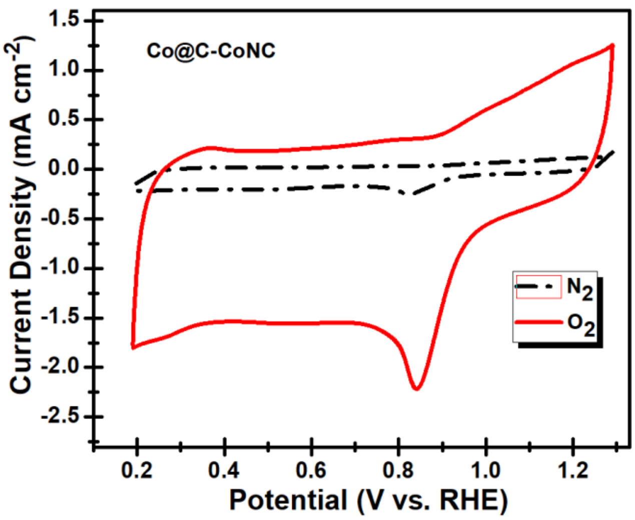

Fig. S17 CV curves for Co@C-CoNC catalyst tested in 0.1 M KOH electrolyte

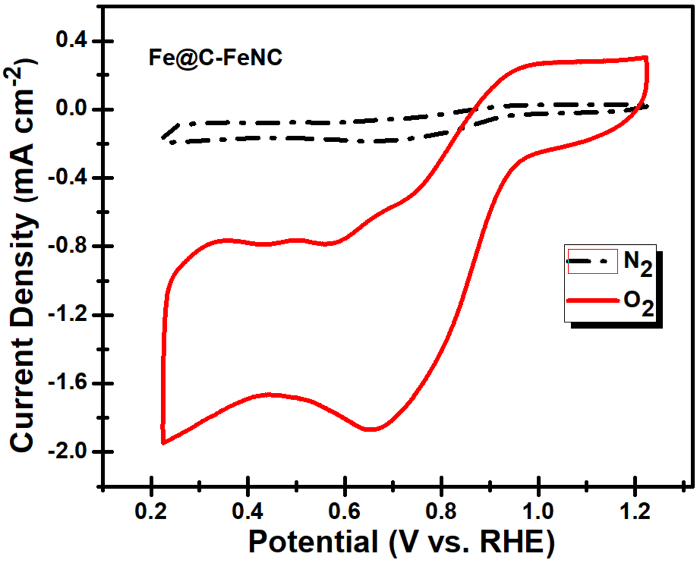

Fig. S18 CV curves for Fe@C-FeNC catalyst tested in 0.1 M KOH electrolyte

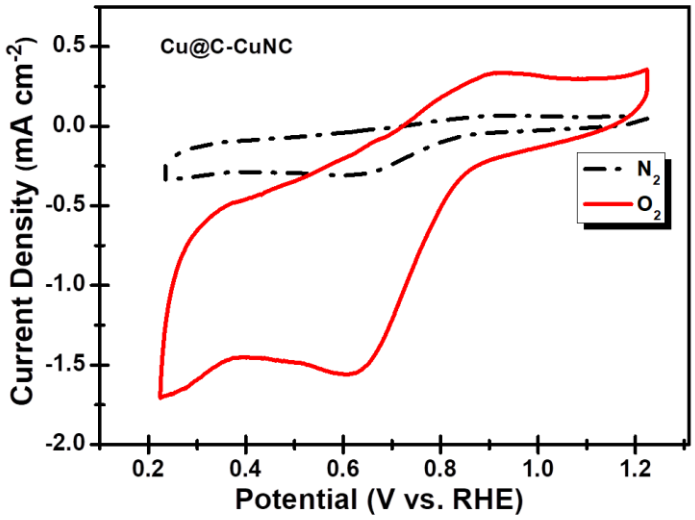

Fig. S19 CV curves for Cu@C-CuNC catalyst tested in 0.1 M KOH electrolyte

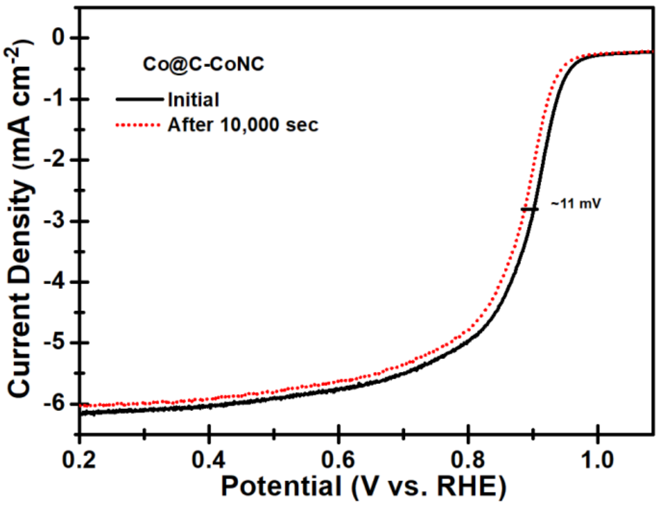

Fig. S20 ORR LSV polarization curves for Co@C-CoNC after durability test in 0.1 M KOH electrolyte

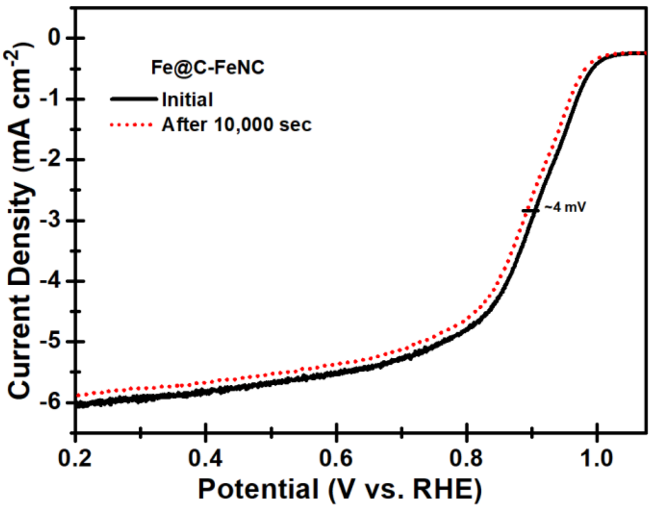

Fig. S21 ORR LSV polarization curves for Fe@C-FeNC after durability test in 0.1 M KOH electrolyte

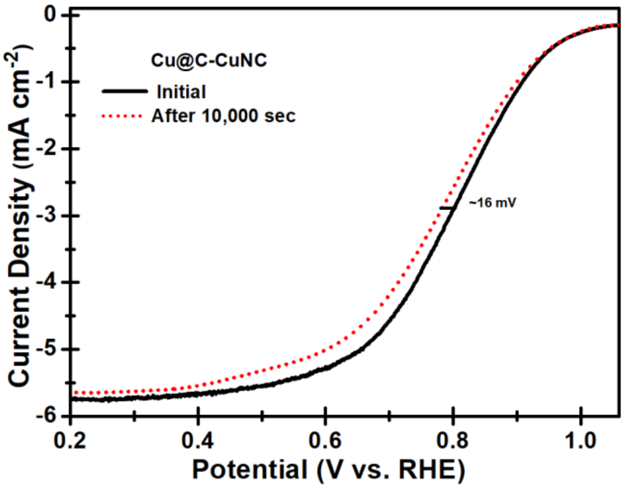

Fig. S22 ORR LSV polarization curves for Cu@C-CuNC after durability test in 0.1 M KOH electrolyte

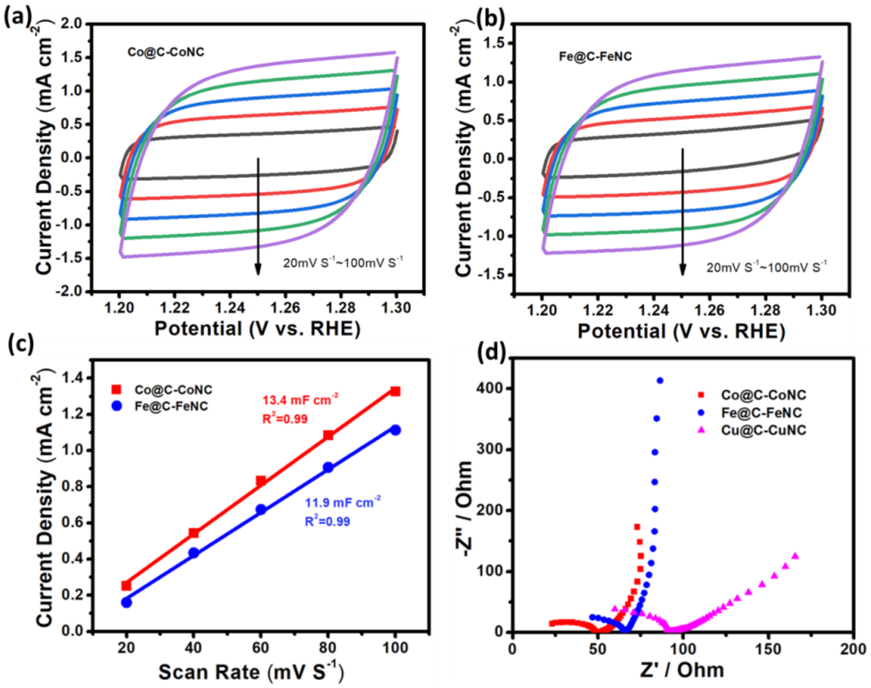

Fig. S23 a-b CV curves, and their corresponding. **c** CdI at different scan rates, and **d** EIS spectra of Co@C-CoNC, and Fe@C-FeNC catalysts in 0.1 M KOH electrolyte

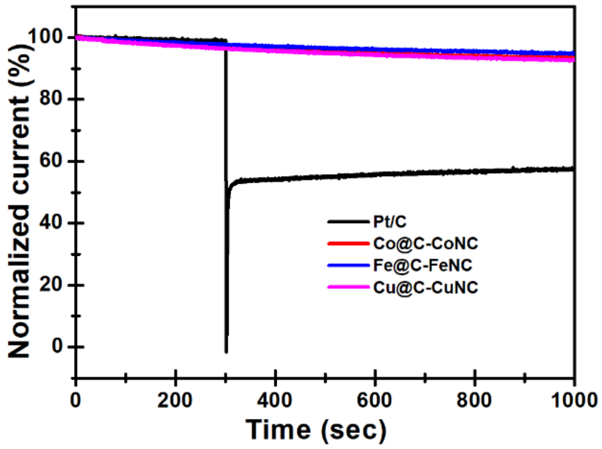

Fig. S24 Methanol tolerance test (3.0 M methanol injected at ~300 s) for prepared catalysts in comparison with benchmark Pt/C

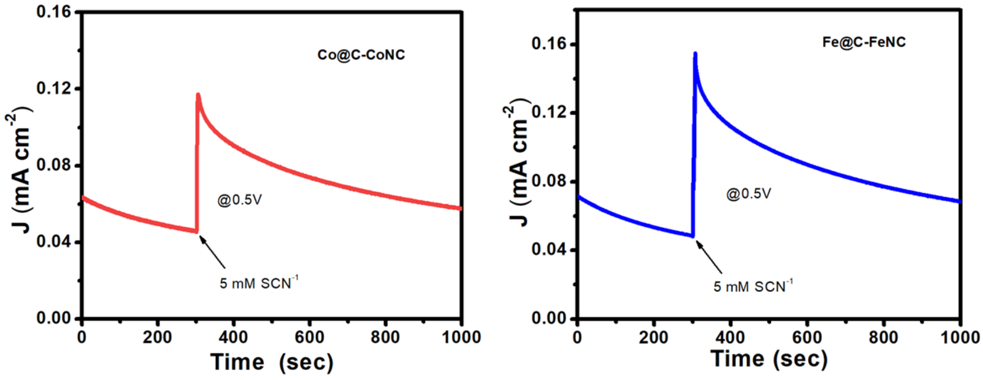

Fig. S25 Cyanide poisoning test on Co@C-CoNC and Fe@C-FeNC catalysts

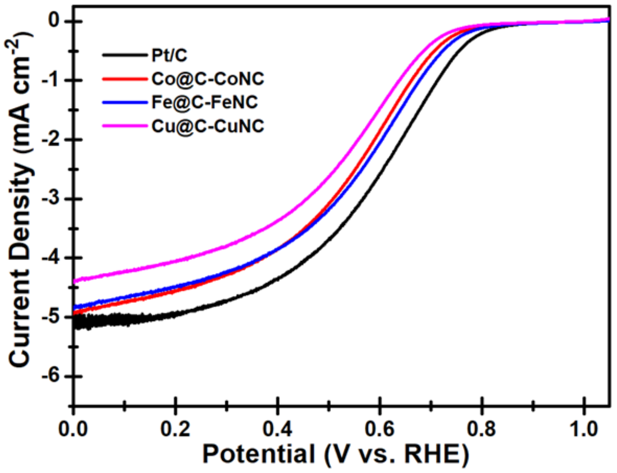

Fig. S26 ORR LSV curves of Co@C-CoNC, Fe@C-FeNC, Cu@C-CuNC, and Pt/C tested in 0.1 M $HClO_4$ electrolyte.

Table S3 Summarized electrocatalytic ORR performance of some previously reported robust catalysts with the present work

**Electrocatalyst**	**Electrolyte**	**ORR ($E_{1/2}$; V vs. RHE)**	**Tafel value (mV dec^{-1})**	**Refs.**
Co@C-CoNC	0.1 M KOH	**0.906**	65	***This work***
Fe@C-FeNC	0.1 M KOH	**0.917**	64	***This work***
Cu@C-CuNC	0.1 M KOH	**0.829**	71	***This work***
Mg-N-C	0.1 M KOH	0.910	62	[S17]
Co SAs/3D GFs	0.1 M KOH	0.901	71	[S18]
Fe–N–C	0.1 M KOH	0.846	-	[S19]
Fe2-N-C	0.1 M KOH	0.905	83	[S20]
Fe–N–C (900)	0.1 M KOH	0.927	71.8	[S21]
Fe1/d-CN	0.1 M KOH	0.950	54	[S22]
Co-SAs/SNPs@NC	0.1 M KOH	0.898	65	[S23]
$Cu_{6.81}$–CoFS	0.1 M KOH	0.800	88	[S24]
Co@IC/MoC@PC	0.1 M KOH	0.875	78	[S25]
Cu–N–C	0.1 M KOH	0.850	37	[S26]
FeCo SAs@Co/N-GC	0.1 M KOH	0.880	49.1	[S27]
MCN	0.1 M KOH	0.800	59	[S28]
Fe SA-NSC-900	0.1 M KOH	0.860	59	[S29]
Fe-N-HMCTs	0.1 M KOH	0.872	89	[S30]
FeSAC-N-C	0.1 M KOH	0.870	60	[S31]
PNGF	0.1 M KOH	0.845	—	[S32]
$Mn_{0.5}(Fe_{0.3}Ni_{0.7})_{0.5}$/MWCNT	0.1 M KOH	0.841	—	[S33]
NCN-1000-5	0.1 M KOH	0.830	86	[S34]
W_2N/WC	0.1 M KOH	0.810	58.13	[S35]
Fe_3C-Co/NC	0.1 M KOH	0.885	—	[S36]
Co/CNFs (1000)	0.1 M KOH	0.896	73	[S37]
Co_2P/CoN-in-NCNTs	0.1 M KOH	0.850	49	[S38]
Mo-CN/C@MoS_2	0.1 M KOH	0.810	—	[S39]
Zn/CoNC	0.1 M KOH	0.861	67	[S40]
Ni-NHGF	0.1 M KOH	0.820	—	[S5]
Meso-CoNC@GF	0.1 M KOH	0.870	75.7	[S41]

Table S4 Summarized electrocatalytic OER performance of some previously reported robust catalysts with the present work

Electrocatalyst	Electrolyte	OER (E_{J10}; V vs. RHE)	Tafel value (mV dec^{-1})	Refs.
Co@C-CoNC	0.1 M KOH	**1.638**	73	***This work***
Fe@C-FeNC	0.1 M KOH	**1.748**	151	***This work***
MCN	0.1 M KOH	1.650	69	[S28]
NCN-1000-5	0.1 M KOH	1.640	146	[S34]
Fe_3C-Co/NC	0.1 M KOH	1.601	—	[S36]
Co/CNFs (1000)	1.0 M KOH	1.640	79	[S37]
Co_2P/CoN-in-NCNTs	0.1 M KOH	1.650	-	[S38]
$NiFe_2O_4$(QDs)/CNTs	0.1 M KOH	1.680		[S42]
Meso-CoNC@GF	0.1 M KOH	1.660	201.3	[S41]
Co-NC@Al_2O_3	0.1 M KOH	1.647	47.8	[S43]
Mo–N/C@MoS_2	0.1 M KOH	1.620	72	[S39]
Fe–N_x–C	0.1 M KOH	1.830	243	[S44]
$Ni_{66}Fe_{34}$-NC	0.1 M KOH	1.699	120	[S45]

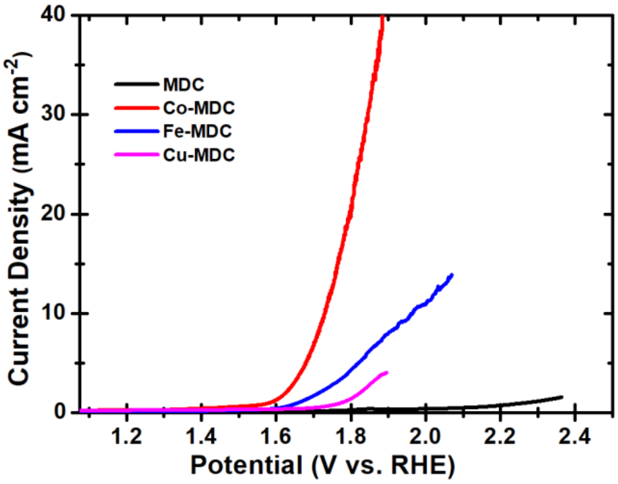

Fig. S27 OER polarization curves for the bare MDC and M-MDC (M= Co, Fe, and Cu) samples in 0.1 M KOH electrolyte

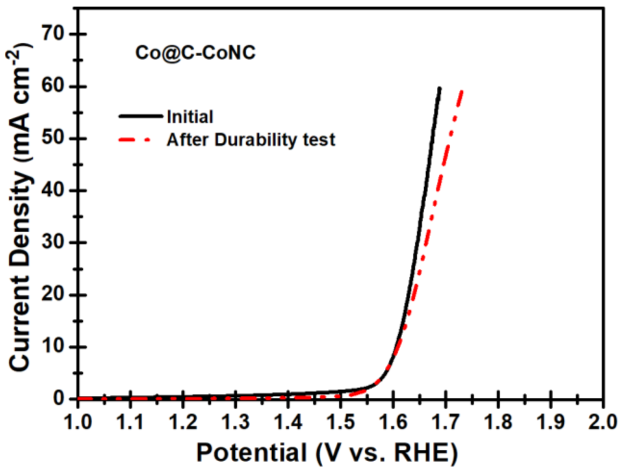

Fig. S28 OER polarization curves for Co@C-CoNC catalyst after durability test in 0.1 M KOH electrolyte

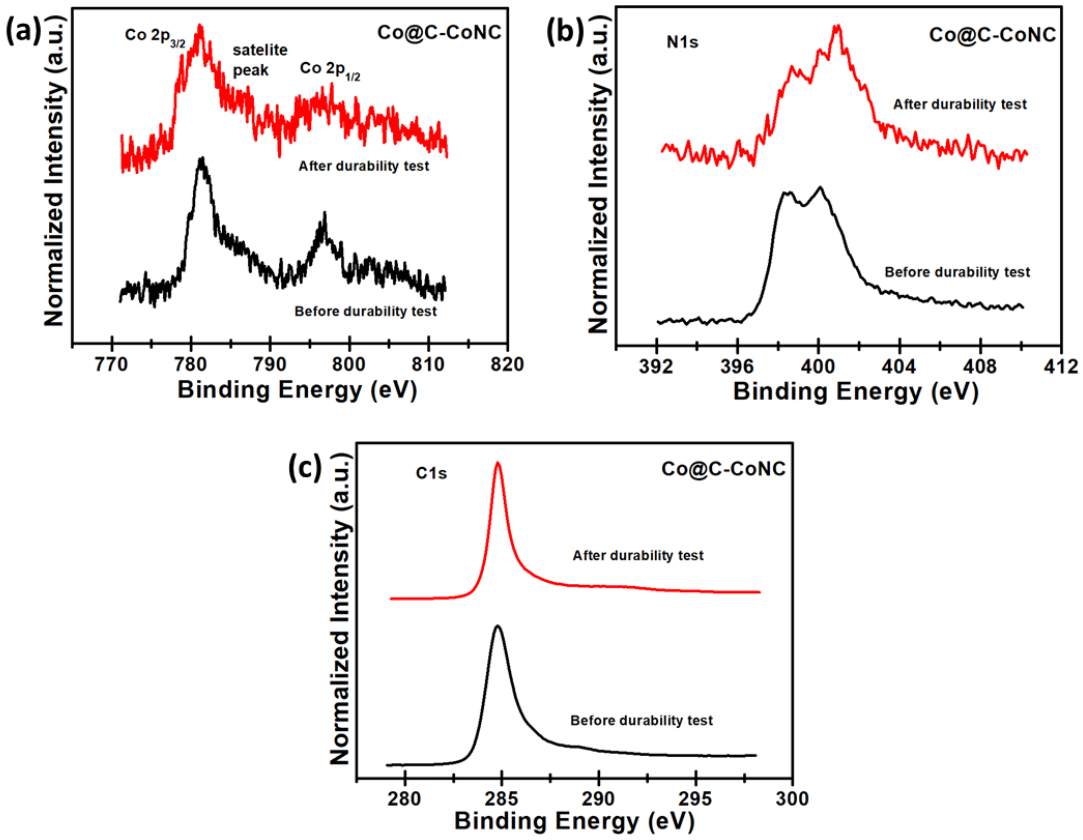

Fig. S29 The core-level XPS spectra of Co2p, N1s, and C1s for Co@C-CoNC (before and after the durability test)

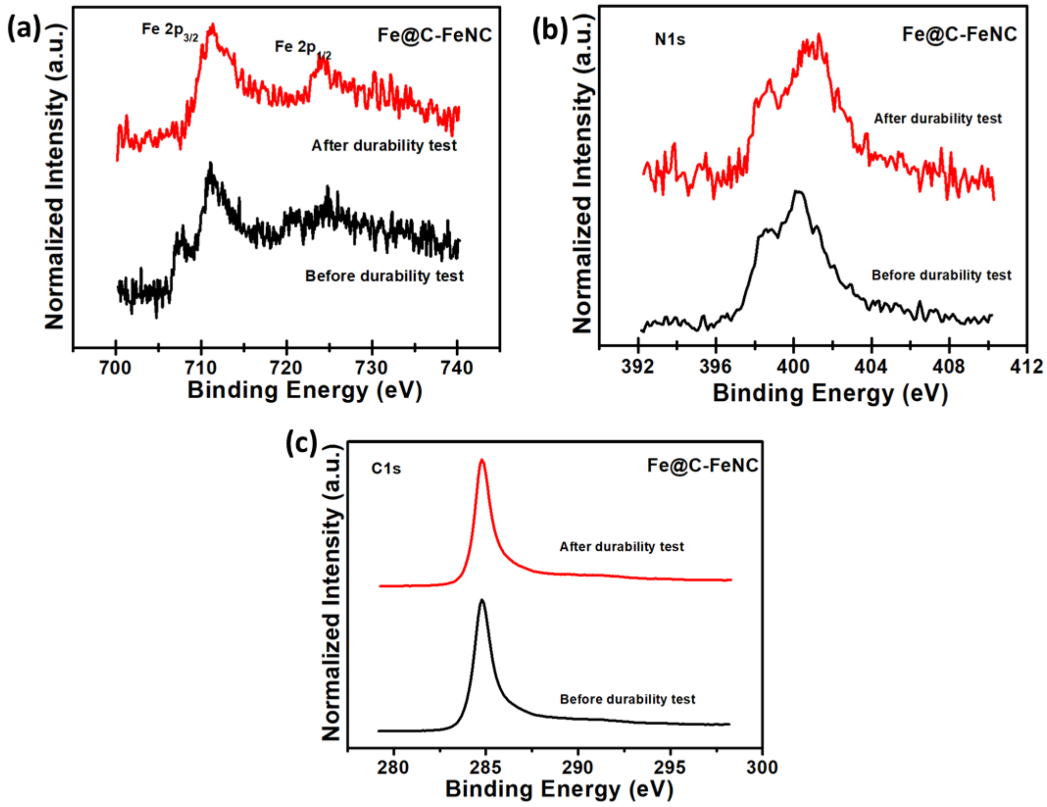

Fig. S30 The core-level XPS spectra of Fe2p, N1s, and C1s for Fe@C-FeNC (before and after the durability test)

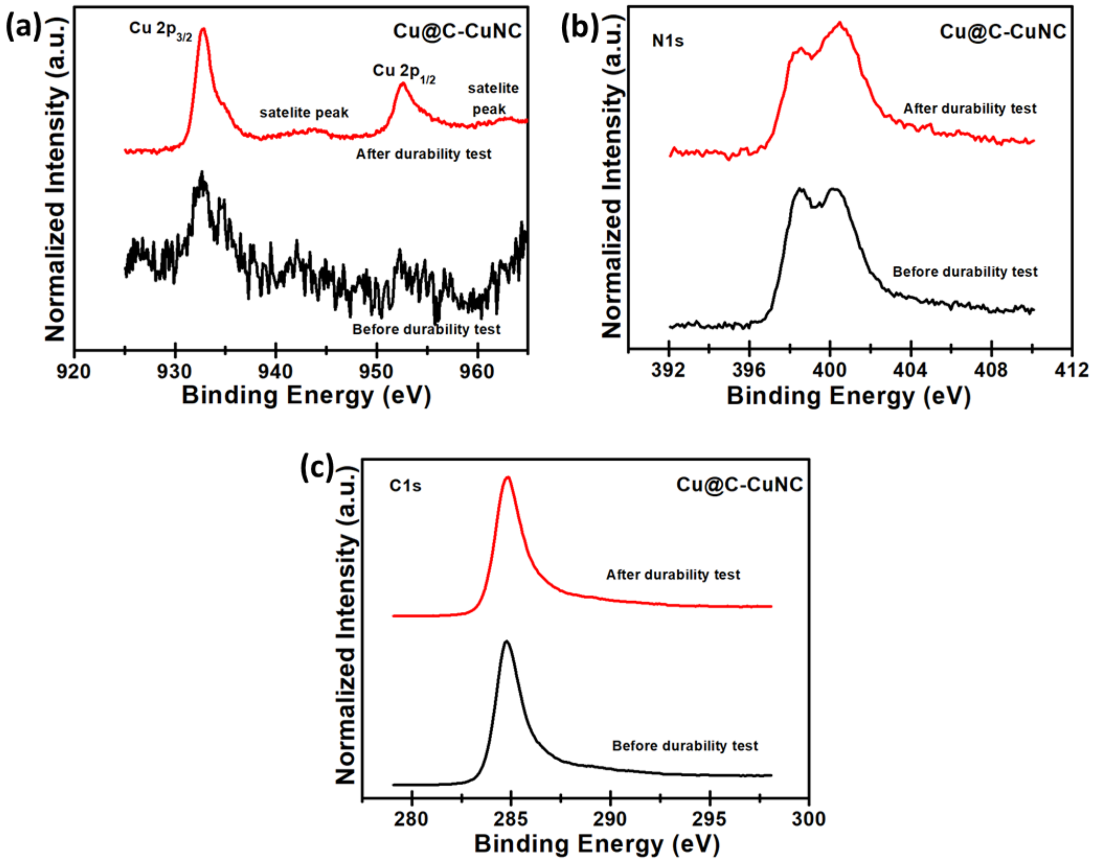

Fig. S31 The core-level XPS spectra of Cu2p, N1s, and C1s for Cu@C-CuNC (before and after the durability test)

Table S5 The chemical compositions of catalysts after the durability test

Catalysts	Carbon (wt%)	Metal (wt%)	Nitrogen (wt%)
Co@C-CoNC	93.50	2.16	4.34
Fe@C-FeNC	94.15	2.54	3.31
Cu@C-CuNC	93.15	1.30	5.55

Table S6 Summarized bi-functional electrocatalytic performance of some previously reported robust catalysts with the present study

Electrocatalyst	ORR ($E_{1/2}$; V *vs.* RHE)	OER (E_{J10};V *vs.* RHE)	△E (E_{J10}- $E_{1/2}$)	Tafel value (mV dec^{-1}) ORR	Tafel value (mV dec^{-1}) OER	Refs.
Co@C-CoNC	0.906	1.638	**0.732**	65	73	***This work***
Fe@C-FeNC	0.917	1.748	**0.831**	64	151	***This work***
$Cu_{6.81}$–CoFS	0.800	1.540	0.740	—	88	[S24]
Ni–N_4/GHSs/Fe–N_4	0.830	1.620	0.790	55	81	[S46]
Co-N,B-CSs	0.830	1.660	0.830	64	—	[S47]
FeN_x-PNC	0.860	1.635	0.775	—	80	[S48]
$Ni_{66}Fe_{34}$-NC	0.850	1.699	0.849	107	120	[S45]
Fe–N_x–C	0.910	1.830	0.920	69	243	[S44]
Co–N–PDEB	0.840	1.920	1.080	—	153	[S49]
Mn/Co–N–C	0.800	1.660	0.860	77	145	[S50]
CoN_4/NG	0.870	1.610	0.740	70	—	[S51]
Ni|MnO/CNF	0.826	1.589	0.763	—	71.5	[S52]

Na_2CoPO_4F	0.792	1.647	0.855	105	—	[S53]
MCN	0.800	1.650	0.850	59	69	[S28]
Co_4N/CNW/CC	0.800	1.540	0.740	—	81	[S54]
PNGF	0.845	1.550	0.705	—	—	[S32]
$Mn_{0.5}(Fe_{0.3}Ni_{0.7})_{0.5}$/MWCNT	0.840	1.570	0.730	—	—	[S33]
NCN-1000-5	0.830	1.640	0.810	86	146	[S34]
Meso-CoNC@GF	0.870	1.660	0.790	75.7	201.3	[S41]
W_2N/WC	0.810	1.550	0.740	58.13	94.50	[S35]
Co_2P/CoN-in-NCNTs	0.850	1.650	0.800	49	-	[S38]
Mo-N/C@MoS_2	0.810	1.620	0.810	—	72	[S39]
Ni-NHGF	0.820	1.580	0.760	—	63	[S5]
$NiFe_2O_4$(QDs)/CNTs	0.780	1.680	0.900	50	—	[S42]
NiCo/NLG©\270	0.820	1.570	0.750	—	—	[S55]
Fe_3C-Co/NC	0.885	1.601	0.712	—	—	[S36]

Table S7 Comparison of rechargeable ZAB performance of the present study with previously reported robust catalysts used for ZABs

**Catalyst**	**Electrolyte**	**Power density mW cm^{-2}**	**Refs.**
Co@C-CoNC	6 M KOH	**162.80**	***This work***
CoNi-SAs/NC	6 M KOH	101.40	[S56]
Co/Co-N-C	6 M KOH	132.00	[S57]
Co/CNFs (1000)	18 M KOH	130.00	[S37]
Meso-CoNC@GF	6 M KOH	154.40	[S41]
Co-NC@Al_2O_3	11.25 M KOH	72.40	[S43]
Pt-SCFP/C-12	6 M KOH	122.00	[S58]
Pd/FeCo	6 M KOH	117.00	[S59]
Fe–N_x–C	6 M KOH	96.40	[S44]
NiCo/NLG-270	6 M KOH	103.00	[S55]
$Cu_{6.81}$–CoFS	6 M KOH	100.00	[S24]
Fe1/d-CN	6 M KOH	78.00	[S22]

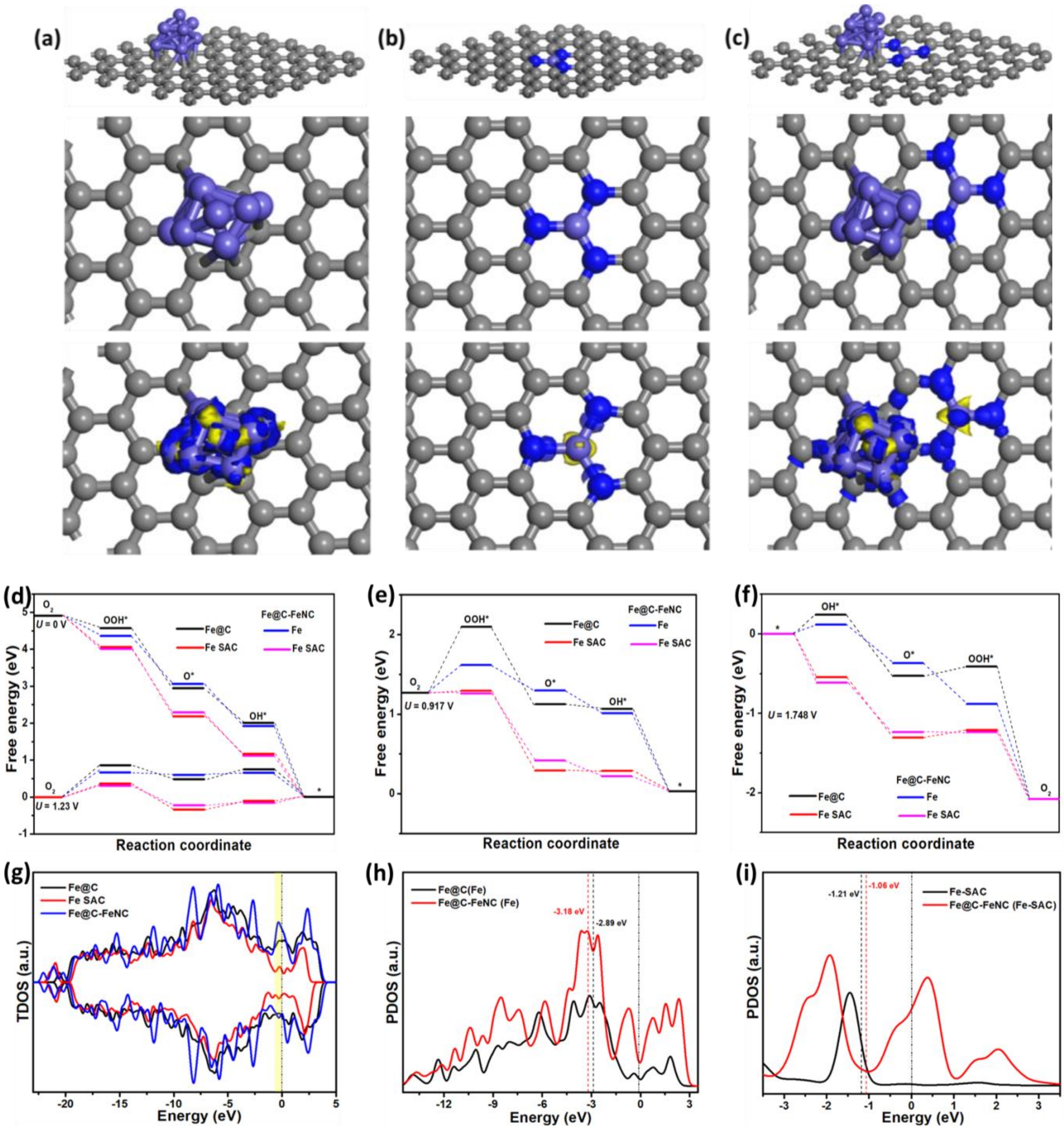

Fig. S32 Atomic models with charge density difference plots for **a** Fe@C, **b** Fe SAC (i.e., Fe–N_3), and **c** Fe NP and adjacent to Fe SACs in Fe@C- FeNC, whereas yellow and blue region represents electron accumulation and depleted regions respectively. **d-f** Free energy diagrams for ORR and OER, while the black line represents Fe NP in bare Fe@C, red lines represent Fe SAC in Fe-N_3, blue and magenta lines represent the Fe NP and adjacent to Fe SACs in Fe@C-FeNC respectively. **g-f** Total and projected electron density of states (on d orbitals of Fe) for bare Fe@C, Fe SAC, and Fe@C-FeNC catalysts

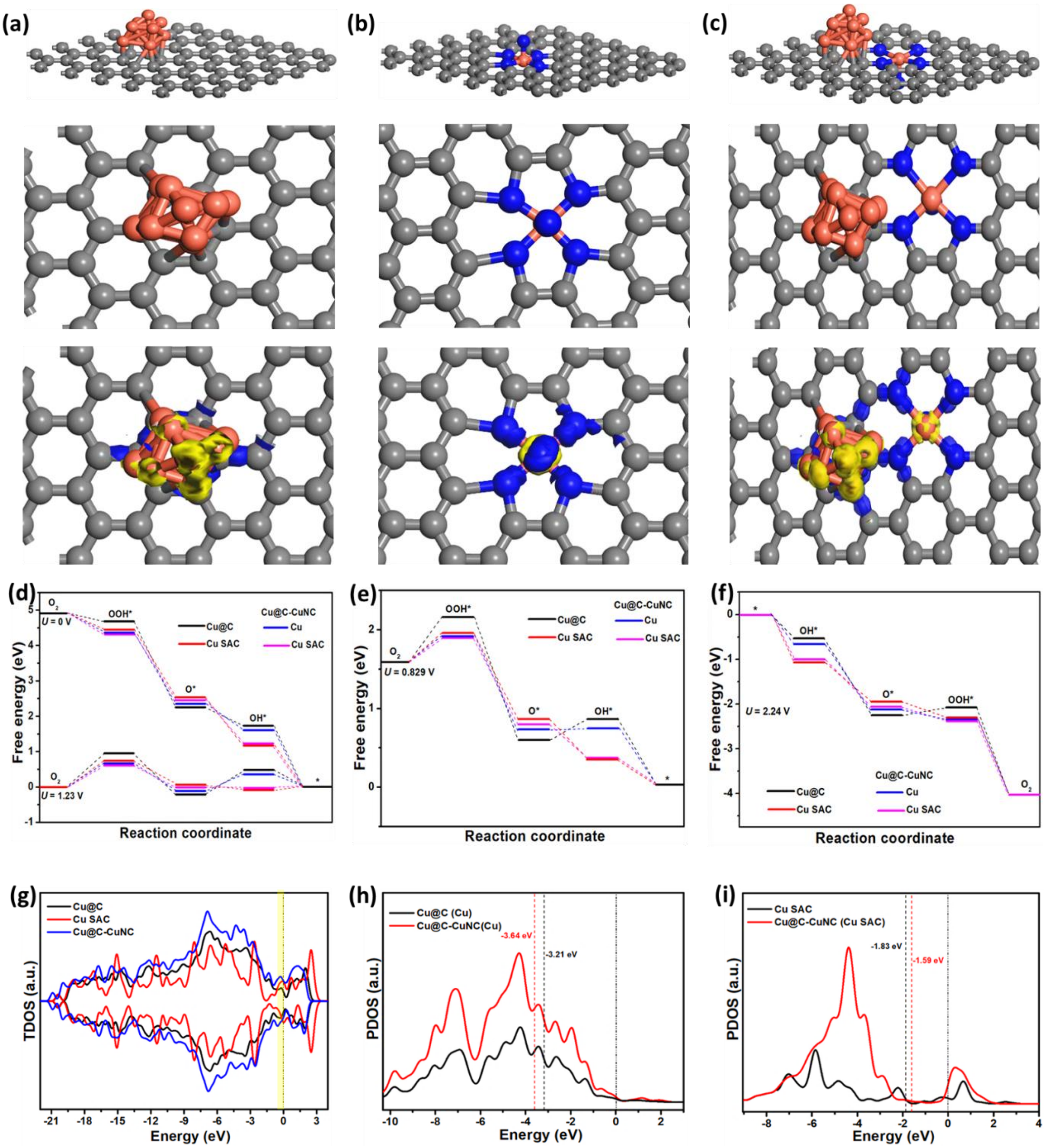

Fig. S33 Atomic models with charge density difference plots for **a** Cu@C, **b** Cu SAC (i.e., Cu-N_5), and **c** Cu NP and adjacent to Cu SACs in Cu@C-CuNC, whereas yellow and blue region represents electron accumulation and depleted regions respectively. **d-f** Free energy diagrams for ORR and OER, while the black line represents Cu NP in bare Cu@C, red lines represent Cu SAC in Cu-N_5, blue and magenta lines represent the Cu NP and adjacent to Cu SACs in Cu@C-CuNC respectively. **g-f** Total and projected electron density of states (on d orbitals of Cu) for bare Cu@C, Cu SAC, and Cu@C-CuNC catalysts

Supplementary References

[S1] O.V. Dolomanov, L.J. Bourhis, R.J. Gildea, J.A. Howard, H. Puschmann, OLEX2: a complete structure solution, refinement and analysis program. J. Appl. Cryst. **42**(2), 339-341 (2009). https://doi.org/10.1107/S0021889808042726

[S2] G.M. Sheldrick, SHELXT–integrated space-group and crystal-structure determination. Acta Cryst. **71**(1), 3-8 (2015). https://doi.org/10.1107/S2053273314026370

[S3] G.M. Sheldrick, Crystal structure refinement with SHELXL. Acta Crystallogr. C Struct. Chem. **71**(1), 3-8 (2015). https://doi.org/10.1107/S2053229614024218

[S4] S. Chandrasekaran, D. Ma, Y. Ge, L. Deng, C. Bowen et al., Electronic structure engineering on two-dimensional (2D) electrocatalytic materials for oxygen reduction, oxygen evolution, and hydrogen evolution reactions. Nano Energy **77**, 105080 (2020). https://doi.org/10.1016/j.nanoen.2020.105080

[S5] H. Fei, J. Dong, Y. Feng, C.S. Allen, C. Wan et al., General synthesis and definitive structural identification of MN_4C_4 single-atom catalysts with tunable electrocatalytic activities. Nat. Catal. **1**(1), 63-72 (2018). https://doi.org/10.1038/s41929-017-0008-y

[S6] Y. He, Q. Shi, W. Shan, X. Li, A.J. Kropf et al., Dynamically unveiling metal–nitrogen coordination during thermal activation to design high-efficient atomically dispersed CoN_4 active sites. Angew. Chem. Int. Ed. **60**(17), 9516-9526 (2021). https://doi.org/10.1002/anie.202017288

[S7] X. Wei, S. Song, N. Wu, X. Luo, L. Zheng et al., Synergistically enhanced single-atomic site Fe by Fe_3C@C for boosted oxygen reduction in neutral electrolyte. Nano Energy **84**,105840 (2021). https://doi.org/10.1016/j.nanoen.2021.105840

[S8] H. Xu, D. Cheng, D. Cao, X.C. Zeng, A universal principle for a rational design of single-atom electrocatalysts. Nat. Catal. **1**(5), 339-348 (2018). https://doi.org/10.1038/s41929-018-0063-z

[S9] X. Wei, X. Luo, N. Wu, W. Gu, Y. Lin et al., Recent advances in synergistically enhanced single-atomic site catalysts for boosted oxygen reduction reaction. Nano Energy **84,** 105817 (2021). https://doi.org/10.1016/j.nanoen.2021.105817

[S10] Z. Zhang, J. Cai, H. Zhu, Z. Zhuang, F. Xu et al., Simple construction of ruthenium single atoms on electrospun nanofibers for superior alkaline hydrogen evolution: a dynamic transformation from clusters to single atoms. Chem. Eng. J. **392,** 123655 (2020). https://doi.org/10.1016/j.cej.2019.123655

[S11] S. Chandrasekaran, N. Li, Y. Zhuang, L. Sui, Z. Xiao et al., Interface charge density modulation of a lamellar-like spatially separated Ni_9S_8 nanosheet/Nb_2O_5 nanobelt heterostructure catalyst coupled with nitrogen and metal (M = Co, Fe, or Cu) atoms to accelerate acidic and alkaline hydrogen evolution reactions. Chem. Eng. J. **431**, 134073 (2022). https://doi.org/10.1016/j.cej.2021.134073

[S12] W. Liu, P. Geng, S. Li, R. Zhu, W. Liu et al., Self-supported three-dimensional WP_2 (WP) nanosheet arrays for efficient electrocatalytic hydrogen evolution. Int. J. Hydrog. Energy **45**(53), 28576-28585 (2020). https://doi.org/10.1016/j.ijhydene.2020.07.144

[S13] C.Y. Su, H. Cheng, W. Li, Z.Q. Liu, N. Li et al., Atomic modulation of FeCo–nitrogen–carbon bifunctional oxygen electrodes for rechargeable and flexible all-solid-state zinc–air battery. Adv. Energy Mater. **7**(13), 1602420 (2017). https://doi.org/10.1002/aenm.201602420

[S14] K. Ding, J. Hu, J. Luo, L. Zhao, W. Jin et al., Robust electronic correlation of Co-CoN_4 hybrid active sites for durable rechargeable Zn-air batteries. Adv. Funct. Mater. **32**(52), 2207331, (2022). https://doi.org/10.1002/adfm.202207331

[S15] R. Ma, G. Lin, Y. Zhou, Q. Liu, T. Zhang et al., A review of oxygen reduction mechanisms for metal-free carbon-based electrocatalysts. npj Comput. Mater. **5**(1), 78 (2019). https://doi.org/10.1038/s41524-019-0210-3

[S16] C. Sun, J. Ding, H. Wang, J. Liu, X. Han et al., Cobalt sulfides constructed heterogeneous interfaces decorated on N,S-codoped carbon nanosheets as a highly efficient bifunctional oxygen electrocatalyst. J. Mater. Chem. A **9**(24), 13926-13935 (2021). https://doi.org/10.1039/d1ta02330f

[S17] S. Liu, Z. Li, C. Wang, W. Tao, M. Huang et al., Turning main-group element magnesium into a highly active electrocatalyst for oxygen reduction reaction. Nat. Commun. **11**, 938 (2020). https://doi.org/10.1038/s41467-020-14565-w

[S18] H. Zhou, T. Yang, Z. Kou, L. Shen, Y. Zhao et al., Negative pressure pyrolysis induced highly accessible single sites dispersed on 3D graphene frameworks for enhanced oxygen reduction. Angew. Chem. Int. Ed. **59**(46), 20465-20469 (2020). https://doi.org/10.1002/anie.202009700

[S19] H. Adabi, A. Shakouri, N.U. Hassan, J.R. Varcoe, B. Zulevi et al., High-performing commercial Fe–N–C cathode electrocatalyst for anion-exchange membrane fuel cells. Nat. Energy **6**(8), 834-843 (2021). https://doi.org/10.1038/s41560-021-00878-7

[S20] W. Ye, S. Chen, Y. Lin, L. Yang, S. Chen et al., Precisely tuning the number of Fe atoms in clusters on N-doped carbon toward acidic oxygen reduction reaction. Chem **5**(11), 2865-2878 (2019). https://doi.org/10.1016/j.chempr.2019.07.020

[S21] C. Zhu, Q. Shi, B.Z. Xu, S. Fu, G. Wan et al., Hierarchically porous M–N–C (M = Co and Fe) single-atom electrocatalysts with robust MN_x active moieties enable enhanced ORR performance. Adv. Energy Mater. **8**(29), 1801956 (2018). https://doi.org/10.1002/aenm.201801956

[S22] M. Zhao, H. Liu, H. Zhang, W. Chen, H. Sun et al., A pH-universal ORR catalyst with iron single-atom sites derived from double-layer MOF: superior flexible quasi-solid-state rechargeable Zn-air batteries. Energy Environ. Sci. **14,** 6455-6463 (2021). https://doi.org/10.1039/d1ee01602d

[S23] Z. Wang, C. Zhu, H. Tan, J. Liu, L. Xu et al., Understanding the synergistic effects of cobalt single atoms and small nanoparticles: enhancing oxygen reduction reaction catalytic activity and stability for zinc-air batteries. Adv. Funct. Mater. **31**(45), 2104735 (2021). https://doi.org/10.1002/adfm.202104735

[S24] Z. Li, Q. Wang, X. Bai, M. Wang, Z. Yang et al., Doping-modulated strain control of bifunctional electrocatalysis for rechargeable zinc–air batteries. Energy Environ. Sci. **14**(9), 5035-5043 (2021). https://doi.org/10.1039/d1ee01271a

[S25] L. Zhang, Y. Zhu, Z. Nie, Z. Li, Y. Ye et al., Co/MoC nanoparticles embedded in carbon nanoboxes as robust trifunctional electrocatalysts for a Zn–air battery and water electrocatalysis. ACS Nano **15**(8), 13399-13414 (2021). https://doi.org/10.1021/acsnano.1c03766

[S26] F. Li, G.F. Han, H.J. Noh, S.J. Kim, Y. Lu et al., Boosting oxygen reduction catalysis with abundant copper single atom active sites. Energy Environ. Sci. **11**(8), 2263-2269 (2018). https://doi.org/10.1039/c8ee01169a

[S27] N.K. Wagh, D.H. Kim, S.H. Kim, S.S. Shinde, J.H. Lee, Heuristic iron–cobalt-mediated robust pH-universal oxygen bifunctional lusters for reversible aqueous and flexible solid-state Zn–air cells. ACS Nano **15**(9), 14683-14696 (2021). https://doi.org/10.1021/acsnano.1c04471

[S28] J. Yan, Y. Wang, Y. Zhang, S. Xia, J. Yu et al., Direct magnetic reinforcement of electrocatalytic ORR/OER with electromagnetic induction of magnetic catalysts. Adv. Mater. **33**(5), 2007525 (2021). https://doi.org/10.1002/adma.202007525

[S29] M. Wang, W. Yang, X. Li, Y. Xu, L. Zheng et al., Atomically dispersed Fe–heteroatom (N, S) bridge sites anchored on carbon nanosheets for promoting oxygen reduction reaction. ACS Energy Lett. **6**(2), 379-386 (2021). https://doi.org/10.1021/acsenergylett.0c02484

[S30] X. Cui, L. Gao, S. Lei, S. Liang, J. Zhang et al., Simultaneously crafting single-atomic Fe sites and graphitic layer-wrapped Fe_3C nanoparticles encapsulated within mesoporous carbon tubes for oxygen reduction. Adv. Funct. Mater. **31**(10), 2009197 (2021). https://doi.org/10.1002/adfm.202009197

[S31] J. Zhang, Y. Zhao, C. Chen, Y.C. Huang, C.L. Dong et al., Tuning the coordination environment in single-atom catalysts to achieve highly efficient oxygen reduction reactions. J. Am. Chem. Soc. **141**(51), 20118-20126 (2019). https://doi.org/10.1021/jacs.9b09352

[S32] G.L. Chai, K. Qiu, M. Qiao, M.M. Titirici, C. Shang et al., Active sites engineering leads to exceptional ORR and OER bifunctionality in P, N co-doped graphene frameworks. Energy Environ. Sci. **10**(5), 1186-1195 (2017). https://doi.org/10.1039/c6ee03446b

[S33] D.M. Morales, M.A. Kazakova, S. Dieckhöfer, A.G. Selyutin, G.V. Golubtsov et al., Trimetallic Mn-Fe-Ni oxide nanoparticles supported on multi-walled carbon nanotubes as high-performance bifunctional ORR/OER electrocatalyst in alkaline media. Adv. Funct. Mater. **30**(6), 1905992 (2020). https://doi.org/10.1002/adfm.201905992

[S34] H. Jiang, J. Gu, X. Zheng, M. Liu, X. Qiu et al., Defect-rich and ultrathin N doped carbon nanosheets as advanced trifunctional metal-free electrocatalysts for the ORR, OER and HER. Energy Environ. Sci. **12**(1), 322-333 (2019). https://doi.org/10.1039/c8ee03276a

[S35] J. Diao, Y. Qiu, S. Liu, W. Wang, K. Chen et al., Interfacial engineering of W_2N/WC heterostructures derived from solid-state synthesis: a highly efficient trifunctional electrocatalyst for ORR, OER and HER. Adv. Mater. **32**(7), 1905679 (2020). https://doi.org/10.1002/adma.201905679

[S36] C.C. Yang, S.F. Zai, Y.T. Zhou, L. Du, Q. Jiang, Fe_3C-Co nanoparticles encapsulated in a hierarchical structure of N-doped carbon as a multifunctional electrocatalyst for ORR, OER and HER. Adv. Funct. Mater. **29**(27), 1901949 (2019). https://doi.org/10.1002/adfm.201901949

[S37] Z. Yang, C. Zhao, Y. Qu, H. Zhou, F. Zhou et al., Trifunctional self-supporting cobalt-embedded carbon nanotube films for ORR, OER and HER triggered by solid diffusion from bulk metal. Adv. Mater. **31**(12), 1808043 (2019). https://doi.org/10.1002/adma.201808043

[S38] Y. Guo, P. Yuan, J. Zhang, H. Xia, F. Cheng et al., Co_2P–Co-N double active centers confined in N-doped carbon nanotube: Heterostructural engineering for trifunctional catalysis toward HER, ORR, OER and Zn–air batteries driven water splitting. Adv. Funct. Mater. **28**(51), 1805641 (2018). https://doi.org/10.1002/adfm.201805641

[S39] I.S. Amiinu, Z. Pu, X. Liu, K.A. Owusu, H.G.R. Monestel et al., Multifunctional Mo–N/C@MoS_2 electrocatalysts for HER, OER, ORR, and Zn–air batteries. Adv. Funct. Mater. **27**(44), 1702300 (2017). https://doi.org/10.1002/adfm.201702300

[S40] Z. Lu, B. Wang, Y. Hu, W. Liu, Y. Zhao et al., An isolated zinc–cobalt atomic pair for highly active and durable oxygen reduction. Angew. Chem. Int. Ed. **58**(9), 2622-2626 (2019). https://doi.org/10.1002/anie.201810175

[S41] S. Liu, M. Wang, X. Sun, N. Xu, J. Liu et al., Facilitated oxygen chemisorption in heteroatom-doped carbon for improved oxygen reaction activity in all-solid-state Zinc–air batteries. Adv. Mater. **30**(4), 1704898 (2018). https://doi.org/10.1002/adma.201704898

[S42] N. Xu, Y. Zhang, T. Zhang, Y. Liu, J. Qiao, Efficient quantum dots anchored nanocomposite for highly active ORR/OER electrocatalyst of advanced metal-air batteries. Nano Energy **57**, 176-185 (2019). https://doi.org/10.1016/j.nanoen.2018.12.017

[S43] L. Zhu, D. Zheng, Z. Wang, X. Zheng, P. Fang et al., A confinement strategy for stabilizing ZIF-derived bifunctional catalysts as a benchmark cathode of flexible all-solid-state zinc–air batteries. Adv. Mater. **30**(45), 1805268 (2018). https://doi.org/10.1002/adma.201805268

[S44] J. Han, X. Meng, L. Lu, J. Bian, Z. Li et al., Single-atom Fe-N_x-C as an efficient electrocatalyst for zinc–air batteries. Adv. Funct. Mater. **29**(41), 1808872 (2019). https://doi.org/10.1002/adfm.201808872

[S45] M. Ma, A. Kumar, D. Wang, Y. Wang, Y. Jia et al., Boosting the bifunctional oxygen electrocatalytic performance of atomically dispersed Fe site via atomic Ni neighboring. Appl. Catal. B Environ. **274**, 119091 (2020). https://doi.org/10.1016/j.apcatb.2020.119091

[S46] J. Chen, H. Li, C. Fan, Q. Meng, Y. Tang et al., Dual single-atomic Ni-N_4 and Fe-N_4 sites constructing janus hollow graphene for selective oxygen electrocatalysis. Adv. Mater. **32**(30), 2003134 (2020). https://doi.org/10.1002/adma.202003134

[S47] Y. Guo, P. Yuan, J. Zhang, Y. Hu, I.S. Amiinu et al., Carbon nanosheets containing discrete Co-N_x-B_y-C active sites for efficient oxygen electrocatalysis and rechargeable Zn–air batteries. ACS Nano **12**(2), 1894-1901 (2018). https://doi.org/10.1021/acsnano.7b08721

[S48] L. Ma, S. Chen, Z. Pei, Y. Huang, G. Liang et al., Single-site active iron-based bifunctional oxygen catalyst for a compressible and rechargeable zinc–air battery. ACS Nano **12**(2), 1949-1958 (2018). https://doi.org/10.1021/acsnano.7b09064

[S49] K. Kim, T. Kang, M. Kim, J. Kim, Three-dimensional entangled and twisted structures of nitrogen doped poly-(1, 4-diethynylbenzene) chain combined with cobalt single atom as a highly efficient bifunctional electrocatalyst. Appl. Catal. B Environ. **275**, 119107 (2020). https://doi.org/10.1016/j.apcatb.2020.119107

[S50] C. Tang, B. Wang, H.F. Wang, Q. Zhang, Defect engineering toward atomic Co–N_x–C in hierarchical graphene for rechargeable flexible solid Zn-air batteries. Adv. Mater. **29**(37), 1703185 (2017). https://doi.org/10.1002/adma.201703185

[S51] L. Yang, L. Shi, D. Wang, Y. Lv, D. Cao, Single-atom cobalt electrocatalysts for foldable solid-state Zn-air battery. Nano Energy **50**, 691-698 (2018). https://doi.org/10.1016/j.nanoen.2018.06.023

[S52] D. Ji, J. Sun, L. Tian, A. Chinnappan, T. Zhang et al., Engineering of the heterointerface of porous carbon nanofiber–supported nickel and manganese oxide nanoparticle for highly efficient bifunctional oxygen catalysis. Adv. Funct. Mater. **30**(13), 1910568 (2020). https://doi.org/10.1002/adfm.201910568

[S53] L. Sharma, R. Gond, B. Senthilkumar, A. Roy, P. Barpanda, Fluorophosphates as efficient bifunctional electrocatalysts for metal–air batteries. ACS Catal. **10**(1), 43-50 (2019). https://doi.org/10.1021/acscatal.9b03686

[S54] F. Meng, H. Zhong, D. Bao, J. Yan, X. Zhang, In situ coupling of strung Co_4N and intertwined N–C fibers toward free-standing bifunctional cathode for robust, efficient, and flexible Zn–air batteries. J. Am. Chem. Soc. **138**(32), 10226-10231 (2016). https://doi.org/10.1021/jacs.6b05046

[S55] X.R. Wang, J.Y. Liu, Z.W. Liu, W.C. Wang, J. Luo et al., Identifying the key role of pyridinic-N–Co bonding in synergistic electrocatalysis for reversible ORR/OER. Adv. Mater. **30**(23), 1800005 (2018). https://doi.org/10.1002/adma.201800005

[S56] X. Han, X. Ling, D. Yu, D. Xie, L. Li et al., Atomically dispersed binary Co-Ni sites in nitrogen-doped hollow carbon nanocubes for reversible oxygen reduction and evolution. Adv. Mater. **31**(49), 1905622 (2019). https://doi.org/10.1002/adma.201905622

[S57] P. Yu, L. Wang, F. Sun, Y. Xie, X. Liu et al., Co nanoislands rooted on Co–N–C nanosheets as efficient oxygen electrocatalyst for Zn–air batteries. Adv. Mater. **31**(30), 1901666 (2019). https://doi.org/10.1002/adma.201901666

[S58] X. Wang, J. Sunarso, Q. Lu, Z. Zhou, J. Dai et al., High-performance platinum-perovskite composite bifunctional oxygen electrocatalyst for rechargeable Zn–air battery. Adv. Energy Mater. **10**(5), 1903271 (2020). https://doi.org/10.1002/aenm.201903271

[S59] F. Pan, Z. Li, Z. Yang, Q. Ma, M. Wang et al., Porous FeCo glassy alloy as bifunctional support for high-performance Zn-air battery. Adv. Energy Mater. **11**(3), 2002204 (2021). https://doi.org/10.1002/aenm.202002204
